# Effects of celastrol on the heart and liver galaninergic system expression in a mouse model of Western-type diet-induced obesity and metabolic dysfunction-associated steatotic liver disease and steatohepatitis

**DOI:** 10.3389/fphar.2025.1476994

**Published:** 2025-02-04

**Authors:** Nikolina Canová, Jana Šípková, Mahak Arora, Zuzana Pavlíková, Tomáš Kučera, Ondřej Šeda, Tijana Šopin, Tomáš Vacík, Ondřej Slanař

**Affiliations:** ^1^ Institute of Pharmacology, First Faculty of Medicine, Charles University and General University Hospital, Prague, Czechia; ^2^ Institute of Histology and Embryology, First Faculty of Medicine, Charles University, Prague, Czechia; ^3^ Department of Anthropology and Human Genetics, Faculty of Science, Charles University, Prague, Czechia; ^4^ Institute of Biology and Medical Genetics, First Faculty of Medicine, Charles University and General University Hospital, Prague, Czechia

**Keywords:** celastrol, fatostatin, galanin receptor, heart, obesity, MASLD, MASH, mouse

## Abstract

**Background:**

The complexity of the galaninergic system is still not fully understood, especially under specific pre-existing comorbidities related to metabolic dysfunction. A plant-derived triterpenoid celastrol was demonstrated to exert a complex effect on the galaninergic system and to have hepatoprotective and anti-obesity properties. However, the exact molecular mechanisms responsible for these effects remain unclear. Specifically, there are no data on the impact of celastrol on the heart and liver galaninergic system. Therefore, this study aimed to investigate the effects of celastrol on the galaninergic system expression in the heart and liver of mice suffering from diet-induced obesity and metabolic dysfunction-associated steatotic liver disease and steatohepatitis (MASLD/MASH).

**Methods:**

The male mice C57BL/6J were fed a Western-type high-fat diet for 16 and 20 weeks to induce obesity and MASLD/MASH. Celastrol was administered along with a specific diet for the last 4 weeks to evaluate its impact on the progression of these conditions. Moreover, the inhibitor of sterol regulatory element-binding protein 1/2 (SREBP1/2), fatostatin, was also tested to compare its influence on the galaninergic system with celastrol.

**Results:**

The study demonstrates that celastrol treatment was safe and led to a reduction in food and energy intake, body fat and liver weights, and MASLD-to-MASH progression and improved glucose tolerance, serum biochemistry markers, and hepatic lipid peroxidation in mice. Quantitative gene expression originally showed significant regulation of galanin and all three of its receptors (GalR1/2/3) in the heart ventricles and only GalR2 in the liver of obese mice. Celastrol influenced the gene expression of galanin receptors: it downregulated *Galr1* in the heart and upregulated *Galr2* in the liver and *Galr3* in the heart ventricles, potentially affecting energy metabolism, oxidative stress, and inflammation. Fatostatin suppressed gene expression of all the detected members of the galaninergic system in the heart ventricles, depicting the role of SREBP in this process.

**Conclusion:**

These findings suggest that celastrol may beneficially modulate the galaninergic system under obesity and MASLD-to-MASH progression, indicating its potential as a therapeutic agent for disorders associated with metabolic dysfunction.

## 1 Introduction

Neuropeptide galanin is an important member of the so-called galaninergic system. Although 4 decades have passed since its discovery (Tatemoto et al., 1983), there are still numerous biological processes where the role of galanin is not yet fully understood (Jiang and Zheng, 2022; Zhu et al., 2022). The described pleiotropic effects of galanin as a neurotransmitter include its involvement in the regulation of sleep and arousal processes, behavioral processes, anxiety, learning and memory, pain and nociception, and other processes. The galaninergic system has also been found to play an important role in many peripheral organ functions, specifically in the heart and cardiovascular system, pancreas, and gastrointestinal system, as well as in bone, connective tissue, and skin (Lang et al., 2015; Šípková et al., 2017a). The diverse effects of galanin are evident not only in typical physiological conditions but also in pathological contexts (Gopalakrishnan et al., 2021).

The pleiotropy and complexity of galanin-mediated signalization are based on the existence of three different G-protein-coupled receptors (GPCRs), namely, GalR1, GalR2, and GalR3, which transduce the biological signal through different pathways (Jiang and Zheng, 2022). In addition, new ligands with partial homology to the galanin molecule were discovered over the years: GALP (galanin-like peptide) and alarin. According to the current knowledge, only GALP is capable of activating galanin receptors, namely, GalR2/GalR3, while alarin is not, despite their partial homology. Specific receptors for alarin are not known (Fang et al., 2020; Abebe et al., 2022). The newest member of the galaninergic system is spexin, a small peptide with pleiotropic functions that can activate human GalR2 and GalR3 receptors (Behrooz et al., 2020).

There are multiple studies describing the important role of the galaninergic system in metabolism, food intake, and obesity. The hypothalamic activity of galanin through GalR1 stimulation leads to increased fat intake. Moreover, there is a capability of stimulating positive feedback, which can lead to excessive fat intake and obesity (Marcos and Coveñas, 2021). This dysregulation may be followed by glucose intolerance, leading to type 2 diabetes mellitus (T2DM) and metabolic syndrome (Fang et al., 2012). Similarly, fat intake and feeding behavior can also be modified by the activity of GALP (Takenoya et al., 2018). Finally, the role in regulating food intake, satiety status, and, subsequently, obesity risk was confirmed for spexin as well (Behrooz et al., 2020). Spexin was also shown to mitigate high-fat diet (HFD)-induced murine hepatic steatosis both *in vivo* and *in vitro* (Jasmine et al., 2016). The complex role of the galanin family peptides and their receptors is also modified by other regulatory pathways and external factors, like acute and chronic stress (Sciolino et al., 2015; Šípková et al., 2017b). The inter-species differences also should not be neglected as the results of experiments in various animal models may not provide consistent results (Kuramochi et al., 2006; Hirako et al., 2017), raising the question of extrapolation of these data to humans.

Multiple studies have confirmed the presence of galanin receptors in the hearts of different vertebrates, including laboratory mice, rats, and guinea pigs. All the types of galanin receptors were discovered in the heart tissue quite early (Wang et al., 1997a; Wang et al., 1997b), but the exact role of galaninergic signalization in the heart is still not fully understood. In guinea pigs, galanin signalization was involved in positive inotropic action and a prolonged effective refractory period (Kocic, 1998). Galaninergic signalization may also be involved in the pathophysiological response to myocardial injury; for example, the myocardial galanin content was increased after cardiac ischemia and reperfusion in rats (Ewert et al., 2008). This phenomenon could be theoretically used for the treatment of cardiac muscle ischemic injury in the future (Pisarenko et al., 2017).

**FIGURE 1:**
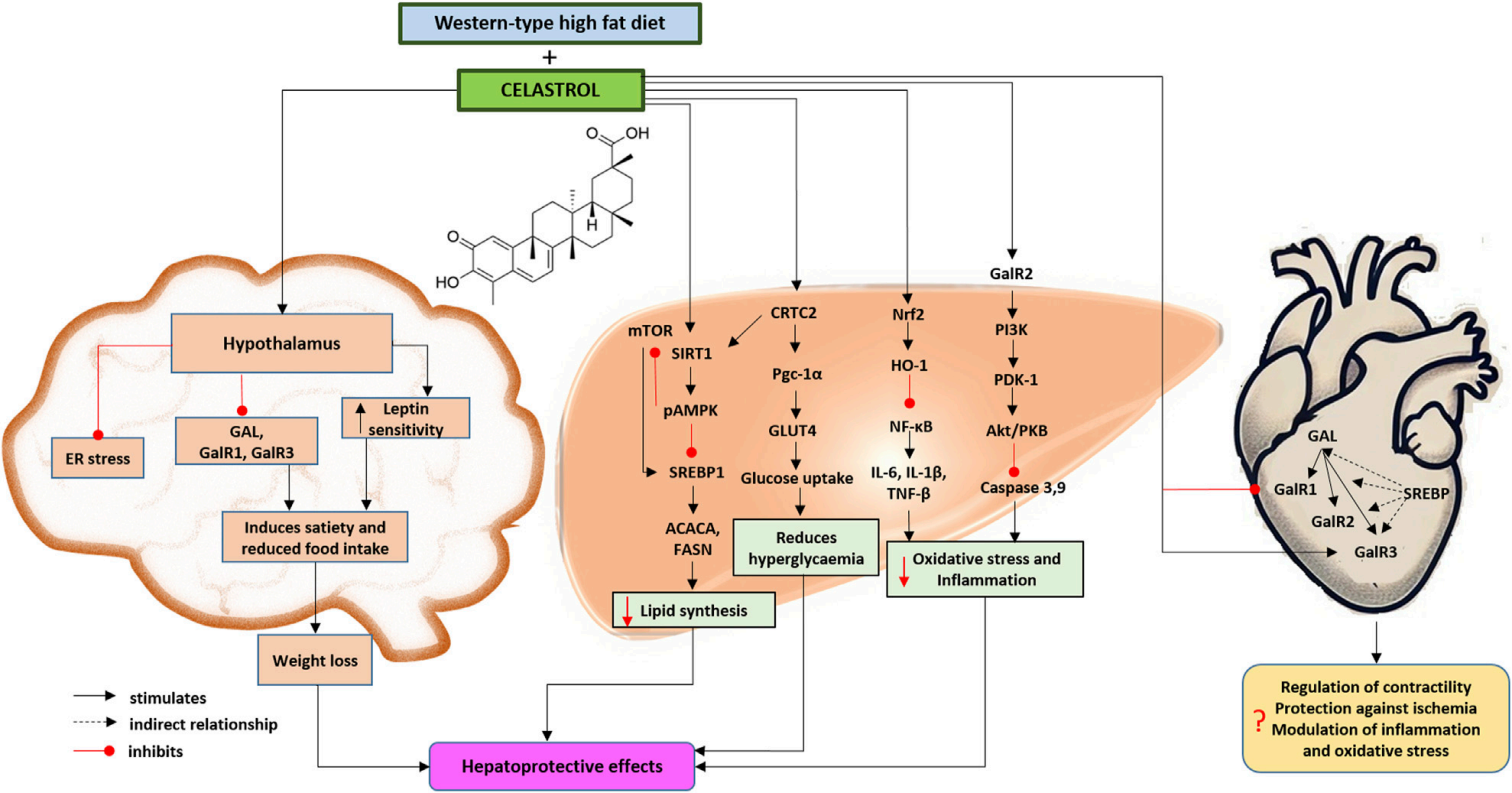
Proposed effects of celastrol on the brain, the heart and liver galaninergic system in a mouse model of Western-type diet-induced obesity and metabolic dysfunction-associated steatotic liver disease and steatohepatitis based on the presented results and herein discussed literature (Chen et al., 2015; Zhang et al., 2016; Fang et al., 2019; Palkeeva et al., 2019; Martinelli et al., 2021; Boal et al., 2022; Li M. et al., 2022; Serebryakova et al., 2023; She et al., 2023). Chemical structural formula of celastrol was drawn by using ChemDraw Professional Software (version 23.1.1.3, 64-bit). The heart was painted by OpenAI (ChatGPT 4o, 2024). Abbreviations: ACACA, acetyl-coenzyme A carboxylase alpha; CRTC2, CREB (cAMP response element-binding protein)-regulated transcription coactivator 2; ER, endoplasmic reticulum; FASN, fatty acid synthase; GAL, galanin; GalR1/2/3, galanin receptor 1/2/3; HO-1, heme oxygenase 1; GLUT4, glucose transporter-4; IL-1β, interleukin 1 beta; IL-6, interleukin 6; mTOR, mechanistic target of rapamycin kinase; NF-κB, nuclear factor kappa B; Nrf2, also Nfe2l2, nuclear factor, erythroid derived 2, like 2; pAMPK, phosphorylated adenosine monophosphate-activated protein kinase; PDK-1, phosphoinositide-dependent protein-kinase 1; PKB, protein kinase B; Pgc-1α, peroxisome proliferative activated receptor, gamma, coactivator 1 alpha; PI3K, phosphatidylinositol 3-phosphate; SIRT1, sirtuin 1; SREBP1, sterol regulatory element-binding protein 1; TNF-α, tumor necrosis factor alpha.

Celastrol, 3-hydroxy-9β,13α-dimethyl-2-oxo-24,25,26-trinoroleana-1(10),3,5,7-tetraen-29-oic acid (Figure 1), is a pentacyclic triterpenoid (Cascão et al., 2017). It was isolated from the root of *Tripterygium wilfordii*, which is a plant widely used in traditional Chinese medicine with reported anti-inflammatory and anticancer effects (Lee et al., 2006). When tested as a potential therapeutic agent, it was found that celastrol is a potent leptin sensitizer and anti-obesity substance in mice (Liu et al., 2015; Ye et al., 2024). Wang et al. stated that celastrol promoted white adipose tissue browning and also protected against HFD-induced obesity by the activation of the hypothalamus–sympathetic axis (Wang et al., 2021). Kyriakou et al. found that celastrol-induced weight loss is mediated by the inhibition of leptin-negative regulator protein in the hypothalamus (Kyriakou et al., 2018). Another effect was mentioned by Abu Bakar et al., who found that celastrol interferes with mitochondrial metabolism and increases pyruvate dehydrogenase complex activity while down-regulating pyruvate dehydrogenase kinase 4 expression (Abu Bakar et al., 2022). Moreover, Fang et al. suggested that celastrol leads to weight loss by inhibiting the expression of galanin and GalR1 and GalR3 receptors in the hypothalamus of mice fed on HFD (Fang et al., 2019). In the aforementioned study (Fang et al., 2019), celastrol also led to a decrease in the plasma levels of GALP, indicating that the effect of celastrol on the galaninergic system is quite complex. Finally, celastrol has been shown to have hepatoprotective properties for liver diseases, including metabolic dysfunction-associated fatty liver disease and steatohepatitis (MASLD/MASH) (Li M. et al., 2022). The exact molecular mechanisms that are responsible for these effects remain unclear. Specifically, there are no data on the effect of celastrol on the heart and liver galaninergic system. Several authors have mentioned the possibility of using galanin or the GALP agonist/antagonist in the therapy of obesity and MASH (He et al., 2023). However, the complexity of the galaninergic system is still not fully understood, especially under specific pre-existing comorbidities. Further research is needed for the potential clinical use of galanin and celastrol in the pharmacological treatment of obesity and its related metabolic and cardiovascular diseases in humans.

Therefore, the main goal of this study was to evaluate the effect of celastrol treatment on the heart and liver galaninergic systems in the mouse model of Western-type diet-induced obesity and MASLD/MASH (Figure 1).

## 2 Materials and methods

### 2.1 Materials

Unless otherwise stated, all the high-quality standard chemicals were purchased from Sigma-Aldrich (Merck, Germany) or P-Lab (Czech Republic).

### 2.2 Declaration on experimental animals

All experimental animals were kept under conventional conditions with free access to water and granular food, regulated room temperature, and a 12-hour light regime in an accredited facility of the Institute of Pharmacology and the Center for Experimental Biomodels (CEB) of the First Faculty of Medicine, Charles University, Prague. The work with experimental animals was conducted following the Animal Protection Law of the Czech Republic (501/2020) and the Directive 2010/63/EU of the European Parliament and the Council. It was approved by the Expert Commission for Work with Experimental Animals of the First Faculty of Medicine, Charles University, and the Ministry of Education and Sports of the Czech Republic under the project No. MSMT - 11956/2021-4.

The male mice from inbred strain C57BL/6J (CEB, Prague, Czech Republic) aged at least 5–6 weeks were used for *in vivo* experiments. They were allowed to acclimatize for 1 week before feeding them with a defined diet. All the animals had unlimited access to a specific diet and drinking water. Due to the mutual aggression of the male mice and for the purpose of monitoring the food and fluid intake, the mice were housed individually in separate cages throughout the experiment.

### 2.3 Induction of obesity and metabolic dysfunction-associated fatty liver disease/steatohepatitis in mice and their treatments

A special atherogenic Western-type high-fat diet (WD) in the form of 10-mm pellets (4,575 kcal/kg) containing 1.25% cholesterol (E15723-34, Ssniff Spezialdiäten, Germany, through Anlab, Czech Republic) was used for the induction of obesity and MASLD/MASH, as described previously (Arora et al., 2023). In addition, the mice received fructose (23.1 g/L = 86.62 kcal/L) and sucrose (18.9 g/L = 74.47 kcal/L), FG, in drinking water. The negative control group received a pelleted standard diet (STD; 3,226 kcal/kg; Altromin 1324, from Velaz, Lysolaje, Czech Republic). The access to food was unrestricted in all the studied groups. After the induction period for 12 or 16 weeks, the *in vivo* experiments were performed in two independent sets, namely, set 1 and set 2, representing the early stage and late stage of MASH, respectively (Table 1). The mice on WD/FG of each set were randomly divided into two groups: the positive control group (WD) and the celastrol treatment group (WD+CEL). Mice were then kept on the established diet and concurrently treated intraperitoneally on each second day with either the vehicle (DMSO, 1 mL/kg) or CEL (Tripterin, 20 mg, # HY-13067, MedChemExpress, United States, through Scintila, Czech Republic) at the dose of 200 μg/ml/kg for 4 additional weeks (Table 1).

**TABLE 1:** Induction of MASLD/MASH and division of mice into the experimental groups.

Set (Total duration)	Treatment groups (abbreviation, number of mice)	Induction period (diet)	Treatment (intraperitoneal dose)	Frequency of CEL dosing and treatment period
Set 1 (16 weeks)	Negative control (STD, n = 3)	12 weeks (STD + water)	+ DMSO (1 mL/kg)	Each second day for the next 4 weeks
	Positive control (WD, n = 7)^a^	12 weeks (WD/FG)	+ DMSO (1 mL/kg)	
	CEL treatment (WD + CEL, n = 8)		+ Celastrol (200 μg/ml/kg)	
Set 2 (20 weeks)	Negative control (STD, n = 3)	16 weeks (STD + water)	+ DMSO (1 mL/kg)	
	Positive control (WD, n = 8)	16 weeks (WD/FG)	+ DMSO (1 mL/kg)	
	CEL treatment (WD + CEL, n = 8)		+ Celastrol (200 μg/ml/kg)	

CEL, celastrol; STD, standard diet; WD/FG, Western-type diet with fructose and glucose in drinking water.

^a^ One animal was excluded from all evaluations due to a cyst of the right kidney and severe cirrhosis with hyperbilirubinemia (Supplementary Figure S3).

This study was preceded by a pilot study aiming to identify the MASLD/MASH development and the gene expression of the galanin family system members (galanin, galanin-like peptide, galanin receptor 1, galanin receptor 2, and galanin receptor 3) in mouse heart ventricles depending on the duration of the WD/FG feeding for 12–21 weeks. In this pilot study, we found that, under the given conditions, MASLD in mice begins to transit into MASH at week 12 on a Western diet and that by the end of week 16, MASH is already fully developed (data not shown). Therefore, we determined that the 4-week administration of CEL from week 12 could prevent the progression of MASLD to MASH (i.e., the transition of MASLD to MASH representing the early stage of MASH) and treatment from week 16 rather than the treatment of already-advanced diet-induced MASH (i.e., the late stage of MASH).

Although there is extensive research on the functions of sterol regulatory element-binding proteins (SREBPs) and their impact on lipid metabolism, the direct effect of SREBPs on galanin and galanin receptors is not well-documented in the available literature. Therefore, we evaluated fatostatin (FAT) for comparative analysis to reveal the role of SREBPs in the galaninergic system. Unlike CEL, FAT primarily affects fat metabolism and reduces adipose tissue and hepatic fat accumulation by inhibiting the activation of SREBP1/2 without any direct impact on appetite or food intake (Kamisuki et al., 2009; Zhao et al., 2022). For these purposes, we treated additional mice (n = 8) on WD/FG diet with FAT (an intraperitoneal dose of 10 mg/kg, dissolved in DMSO) each second day during weeks 12–16 (corresponding to set 1 of *in vivo* experiments).

### 2.4 Oral glucose tolerance test

An oral glucose tolerance test (OGTT) was performed 36 h before mouse euthanasia to measure the glucose concentration in the blood of experimental mice, as we described previously (Arora et al., 2023) with minor modifications. OGTTs were conducted on mice that had been fasted for 8 h (Andrikopoulos et al., 2008). In relation to oral glucose application (2 g/kg of body weight), blood samples were collected from the tail vein at 0, 30, 60, and 120 min to measure blood glucose levels using the Accu-Chek^®^ Instant glucometer (Czech Dia, Roche). The area under the curve (AUC) of blood glucose was then calculated using the trapezoidal method (AUC = 1/4 * fasting glycemia + 1/2 * 30-min glycemia + 3/4 * 60-min glycemia + 1/2 * 120-min glycemia) (Li L. et al., 2021).

### 2.5 Blood, heart, liver, and fat sampling

The animals were fasted 12 h before terminal sampling. Initially, mice were anesthetized by intraperitoneal application of a ketamine (100 mg/kg) and xylazine (5 mg/kg) mixture (Bioveta, Czech Republic). Blood was terminally withdrawn by retro-orbital puncture using heparinized glass capillaries. Blood samples were left for 30 min at room temperature to clot and then centrifuged at 4,000 rpm for 10 min at 4°C. Serum aliquots were stored at −20°C until biochemical measurements. The mice were further dissected to extract the hearts, livers, and intra-abdominal and epididymal fat, which were washed in cold phosphate-buffered saline (PBS), dried on sterile gauze, and weighed. If necessary, the heart ventricles and individual liver lobes were separated to be utilized for respective analyses, as described below.

### 2.6 Biochemical analysis

Determination of serum concentrations of lipids (total cholesterol and triglycerides), glucose, albumin, liver enzymes (ALT, alanine transaminase; AST, aspartate transaminase; ALP, alkaline phosphatase), and nitrogen metabolites (urea and creatinine) was performed using the customized commercial kits in the routine Central laboratory of the Institute of Medical Biochemistry and Laboratory Diagnostics of the General University Hospital in Prague. The triglyceride content was estimated in liver caudate lobes exactly by following the instructions in the Triglyceride Quantification Colorimetric/Fluorometric Kit manual (# MAK266-1KT, Sigma-Aldrich, through Merck, Germany) and expressed as micrograms per µg of lysate protein, as measured using the Pierce™ BCA Protein Assay Kit (Thermo Fisher Scientific™).

### 2.7 Determination of liver oxidative stress parameters

First, a liver left lateral lobe was homogenized (10% w/v) in the cooled lysis buffer (0.2 M Tris-HCl, pH 7.4, 0.002 M EDTA-Na_2_, and 0.025 M sucrose) and centrifuged at 4,000 rpm for 15 min at 4°C. Then, the supernatant was used for the analysis of oxidative stress markers. To determine the severity of liver oxidative stress caused by the metabolic disease, total lipid peroxidation was estimated by evaluating conjugated dienes, thiobarbituric acid reactive substances (TBARS), and nitrites, as previously described (Farghali et al., 2009; Arora et al., 2023). The total amount of protein in the liver homogenates was determined using the Bio-Rad protein assay (Bio-Rad, Prague, Czech Republic).

### 2.8 Quantitative RT-PCR for gene expression

Two different qRT-PCR methods were used depending on the tissue collected: heart ventricles or liver lobes. RNA from heart ventricles (right and left together) was isolated using the QIAzol Lysis Reagent (QIAGEN, CA, United States). Extracted RNA was purified using the RNeasy Plus Mini Kit, as per the manufacturer’s protocol. Quantitative and qualitative analyses of RNA for quality determination were performed using the Agilent 2100 Bioanalyzer system (Agilent, CA, United States). The RNA integrity number (RIN) was used as an integrity parameter. Only samples showing RIN above 7.5 were used for further analysis (Schroeder et al., 2006). Total RNA (1 µg) was reverse-transcribed with oligo-dT primers using SuperScript IV (Invitrogen, Carlsbad, CA, United States). For validation, the following sets of TaqMan probes (Thermo Fisher Scientific; Waltham, MA, United States) were used: galanin (*Gal*, TaqMan Assay Mm00439056_m1), galanin-like peptide (*Galp*, TaqMan Assay Mm00626135_m1), galanin receptor 1 (*Galr1*, Mm00433515_m1), galanin receptor 2 (*Galr2*, Mm00726392_s1), and galanin receptor 3 (*Galr3*, Mm00443617_m1). The qRT-PCR reaction was performed in triplicate with TaqMan Gene Expression Master Mix (Applied Biosystems), according to the manufacturer’s protocol (Invitrogen, Carlsbad, CA, United States) using the Applied Biosystems 7900HT Real-Time PCR System. Cycle threshold (Ct) values were normalized using glyceraldehyde-3-phosphate dehydrogenase (*Gapdh*) (TaqMan chemistry, Applied Biosystems) as a standard.

Isolated liver left median lobes were stored in the RNAlater™ stabilization solution and maintained at −20°C for further qRT-PCR analysis. TRI reagent (Sigma-Aldrich, Prague, Czech Republic) was used to homogenize livers that were then treated consequently with chloroform, ice-cold isopropanol, and ice-cold 75% ethanol and centrifuged to obtain RNA pellets (Arora et al., 2023). Concentrations of extracted RNA were measured using a NanoReady Micro UV-Vis Spectrophotometer (LifeReal) and were reverse transcribed using a LunaScript RT SuperMix (New England Biolabs), following the manufacturer’s protocol. Furthermore, cDNA was subjected to quantitative PCR using the CFX Connect™ Real-Time PCR Detection System (Bio-Rad), following the Luna Universal qPCR Master Mix (New England Biolabs) protocol, along with the primers listed in Table 2. The data were analyzed using CFX Maestro™ software (Bio-Rad Laboratories). Relative quantification was performed using Livak’s–Schmittgen’s ∆∆Ct method (Livak and Schmittgen, 2001).

**TABLE 2:** List of the primers used for the qRT-PCR analysis of liver expression of selected genes.

Target gene	Forward primer (5′-3′)	Reverse primer (3′-5′)
*Nrf2*	CGC​CAG​C-TAC​TCC​CAG​GTT	GGATATCCAGGGCAAGCG
*Ppargc1a*	GCT​GGT​TGC​CTG​CAT​GAG​T	CCA​ACC​AGA​GCA​GCA​CAC​TCT
*Srebf1*	ATT​GAG​AAG​CGC​TAC​CGG​TCT	TGT​GCA​CTT​CGT​AGG​GTC​AGG
*Mtor*	GTT​TGT​GGC​TCT​GAA​TGA​CCA​G	GCT​CCT​TGA​TTC​TCC​CAA​TGC
*Crtc2*	CAA​CAA​TGT​CAC​CCA​CCT​TGT​C	GGG​CAA​TCG​CTG​GTC​AGT​AG
*Sirt1*	TCTATGCTCGCCTTGCGG	GAC​ACA​GAG​ACG​GCT​GGA​ACT
*Hmox1*	AGC​CGT​CTC​GAG​CAT​AGC​C	ATCCTGGGGCATGCTGTC
*Acaca*	GCT​TAC​AGG​ATG​GTT​TGG​CCT	CAA​ATT​CTG​CTG​GAG​AAG​CCA​C
*Sod2*	CGC​TTA​CAG​ATT​GCT​GCC​TG	GGT​AGT​AAG​CGT​GCT​CCC​ACA
*Fasn*	TCC​TGG​AAC​GAG​AAC​ACG​ATC​T	GAG​ACG​TGT​CAC​TCC​TGG​ACT​TG
*Tnfa*	GCC​TCT​TCT​CAT​TCC​TGC​TTG​T	CTG​ATG​AGA​GGG​AGG​CCA​TTT
*Il1b*	TGC​CAC​CTT​TTG​ACA​GTG​ATG	TGA​TGT​GCT​GCT​GCG​AGA​TT
*Gal*	GAG​CCT​TGA​TCC​TGC​ACT​GAC	GGG​TCC​AAC​CTC​TCT​TCT​CCT​T
*Galr1*	CTT​ACG​TGG​TGT​GCA​CTT​TCG	GGCAGCCAGGATATGCCA
*Galr2*	GAC​TGT​AGT​AGC​TCA​GGT​AG	CGT​TCA​TTT​CCT​CAT​CTT​CC
*Glar3*	CAC​CAT​GTA​TGC​CAG​CAG​CTT	ACC​GTG​CCG​TAG​TAG​CTT​AGG​T
*Hprt1*	TCA​GTC​AAC​GGG​GGA​CAT​AAA	GGG​GCT​GTA​CTG​CTT​AAC​CAG

Abbreviations**:** *Ppargc1a*, also Pgc-1α, peroxisome proliferative activated receptor, gamma, coactivator 1 alpha; *Crtc2*, CREB, cAMP response element-binding protein, regulated transcription coactivator 2; *Fasn*, fatty acid synthase; *Acaca*, acetyl-coenzyme A carboxylase alpha; *Srebf1*, sterol regulatory element-binding transcription factor 1; *Nrf2*, also Nfe2l2, nuclear factor, erythroid derived 2, like 2; *Mtor*, mechanistic target of rapamycin kinase; *Hmox1*, heme oxygenase 1; *Sirt1*, sirtuin 1; *Sod2*, superoxide dismutase 2, mitochondrial; *Tnfa*, tumor necrosis factor alpha; *Il1b*, interleukin 1 beta; *Gal*, galanin; *Galr1/2/3*, galanin receptor 1/2/3; *Hprt1*, hypoxanthine phosphoribosyltransferase 1.

### 2.9 Western blot

As described previously (Arora et al., 2023), the liver samples were homogenized and lysed in RIPA lysis buffer with added protease and phosphatase inhibitors. Equal amounts of lysate protein, specifically 30 μg, as determined by the Pierce™ BCA Protein Assay Kit (Thermo Scientific™), and PageRuler Prestained Protein Ladder (# 26616, Thermo Fisher Scientific, Czech Republic) were subjected to Mini-PROTEAN®TGX Stain-free™ 4%–20% precast gels (# 456-1093, Bio-Rad, Czech Republic) and then transferred electrophoretically onto a methanol-activated Wet Immobilon E (0.45 µm) nitrocellulose membrane. The membranes were blocked by incubating with Tris-buffered saline containing 5% bovine serum albumin (BSA) and 0.1% sodium azide for 30 min at room temperature. Subsequently, the membranes were incubated with primary antibodies overnight at 4°C. The primary antibodies comprised the rabbit anti-SREBP1 polyclonal antibody (1:1,000 dilution, # PA1-337, Invitrogen, through Thermo Fisher Scientific, Czech Republic), rabbit anti-GalR2 polyclonal antibody (1: 400 dilution, # bs-11527R, Bioss Antibodies, through iBioTech, Czech Republic), rabbit anti-HRPT1 monoclonal antibody (1: 1,000 dilution, # A8783, ABclonal Technology), and rabbit anti-beta2-microglobulin monoclonal antibody (1: 1,000 dilution, # 59035, Cell Signaling Technology™). On the next day, the membranes were washed in TBST buffer and incubated with goat anti-rabbit polyclonal IgG and horseradish peroxidase-conjugated antibody (1: 5,000-1: 15,000 dilutions, # ADI-SAB-300-J, Enzo^®^ Life Sciences, through iBioTech, Czech Republic) in 5% non-fat milk blocking solution at room temperature for 1 h. The protein bands on the membranes were visualized via SuperSignal^®^ West Pico Chemiluminescent Substrate (Thermo Scientific™ through GeneTiCA s.r.o., Prague, Czech Republic) by using the ChemiDoc™ MP imaging system (Bio-Rad, Czech Republic). The band intensities of SREBP1 and GalR2 were quantified using ImageJ software (National Institutes of Health, Bethesda, MD, United States) and normalized to the respective house-keeping protein (HRPT1 and β2-microglobulin) and, subsequently, to the corresponding negative control group.

### 2.10 Histology

The heart and liver tissues were fixed in 4% paraformaldehyde and then embedded in paraffin, cut into 7–8-µm-thick sections, and stained with hematoxylin–eosin (HE) or Sirius red. Liver samples of the right median lobe were scored for MASLD and fibrosis. MASLD was scored according to the grading system specifically established by Liang et al. for rodent MASLD models using samples stained with HE (Liang et al., 2014). In brief, steatosis was determined by analyzing hepatocellular vesicular steatosis, namely, macrovesicular steatosis, microvesicular steatosis, and hepatocellular hypertrophy (each scored 0–3). Macrovesicular and microvesicular steatosis were evaluated separately, according to its severity, based on the percentage of total area affected. Hepatocellular hypertrophy, which is defined as the enlargement of cells to more than 1.5 times the normal diameter of hepatocyte, was also assessed based on the percentage of total area affected as well. Inflammation was assessed by counting the number of inflammatory foci present per field, with a focus being a cluster of five inflammatory cells. Five different fields were counted, and their average was then rated into the four categories (score 0, 1, 2, and 3) (Liang et al., 2014). Two key features of MASH, steatosis (score 0–9) and inflammation (score 0–3), were used to calculate the total MASLD score (ranging from 0 to 12 score). If the total steatosis score was 0, MASLD was not diagnosed, regardless of inflammation. MASLD was diagnosed if steatosis was present. Finally, MASH was diagnosed if both steatosis and any inflammation were observed (Liang et al., 2014). Liver fibrosis was identified using 8-µm slides stained with Sirius red (SR) dye and scored according to Kleiner et al. (2005). This liver fibrosis classification system recognizes five stages, namely, stage 0 (no fibrosis), stages 1A/numerically 1, 1B/1.33, and 1C/1.67 (representing mild and moderate perisinusoidal fibrosis and portal/periportal fibrosis, respectively); stage 2 (both perisinusoidal and portal/periportal fibrosis); stage 3 (bridging fibrosis); and stage 4 (cirrhosis). Liver tissue sections were analyzed using a Leica DMLB microscope equipped with a Leica MC170 HD camera. One representative HE-stained section was scored for steatosis and inflammation (Liang et al., 2014), and one representative SR-stained section was scored for fibrosis in each specimen (Kleiner et al., 2005). To exclude differences in individual subjective scoring, all liver samples were scored by the same trained “blinded” histologist throughout the study (Arora et al., 2023).

Some samples of the heart were immediately fixed in 4% paraformaldehyde, cryoprotected with sucrose, embedded into the optimal cutting temperature compound, frozen at −20°C, and stored until further use. For the indirect immunofluorescence method, 7-µm-thick cryosections were used. After thawing and washing in PBS, non-specific antibody-binding sites were blocked with 5% goat serum in PBS. In a pilot study, we used three different primary antibodies: polyclonal rabbit anti-GalR1 (#AGR-011), anti-GalR2 (#AGR-012), and anti-GalR3 (extracellular) (#AGR-013) (all from Alomone Labs, Israel), to screen for positivity in mouse hearts. Interestingly, immunoreactivity was detected only for GalR1. Therefore, other sections were incubated only with polyclonal rabbit anti-GalR1 antibody (Cat. No. LS-C831302, LS Bio, through EXBIO Prague, Czech Republic) diluted 1: 1,000 in PBS + 1.5% normal goat serum overnight at 4°C. For visualization, a secondary goat anti-rabbit IgG biotin antibody (Agilent) diluted 1:400 in the PBS + 5% normal goat serum was applied to sections for 30 min at room temperature. Visualization was carried out using the avidin-biotinylated peroxidase complex (VECTASTAIN ABC Elite Kit, Vector Laboratories) and finally DAB (Agilent) as a substrate.

### 2.11 Determination of TNF-α

TNF-α was estimated using customized ELISA kits, as described in the Supplementary Material.

### 2.12 *In vitro* MASLD model

The palmitic acid (PA)-induced primary hepatocyte lipotoxicity model, as we introduced previously (Arora et al., 2023), is described in detail in the Supplementary Material.

### 2.13 Statistical analysis

Normal distribution of the data was checked using the Shapiro–Wilk test. To compare the differences between groups, one-way or two-way ANOVA with the *post hoc* Bonferroni test, whenever appropriate, was used. To compare histopathological scores between the STD, WD, and WD+CEL groups, the Kruskal–Wallis test with the *post hoc* Dunn test was used. Student’s *t*-test with adjusted p-values was used for pair-wise comparisons. The results of the variables’ data are expressed as the mean with a respective standard deviation (SD). Unless otherwise indicated in the legend of a specific figure, the numbers (n) of all values scored correspond to the number of mice in each group, exactly as presented in Table 1. The differences were considered statistically significant if *p* < 0.05. In the graphs and the result section of *in vivo* experiments, there are significant differences only between negative (STD) and positive (WD) controls and between positive controls (WD) and CEL treatments (CEL+WD) presented. Statistical analyses and data visualization were performed using GraphPad Prism version 8.0.0 for Windows (GraphPad Software, San Diego, California, United States).

## 3 Results

### 3.1 The effect of celastrol treatment on mouse's body, fat and liver weight, and food intake

**FIGURE 2:**
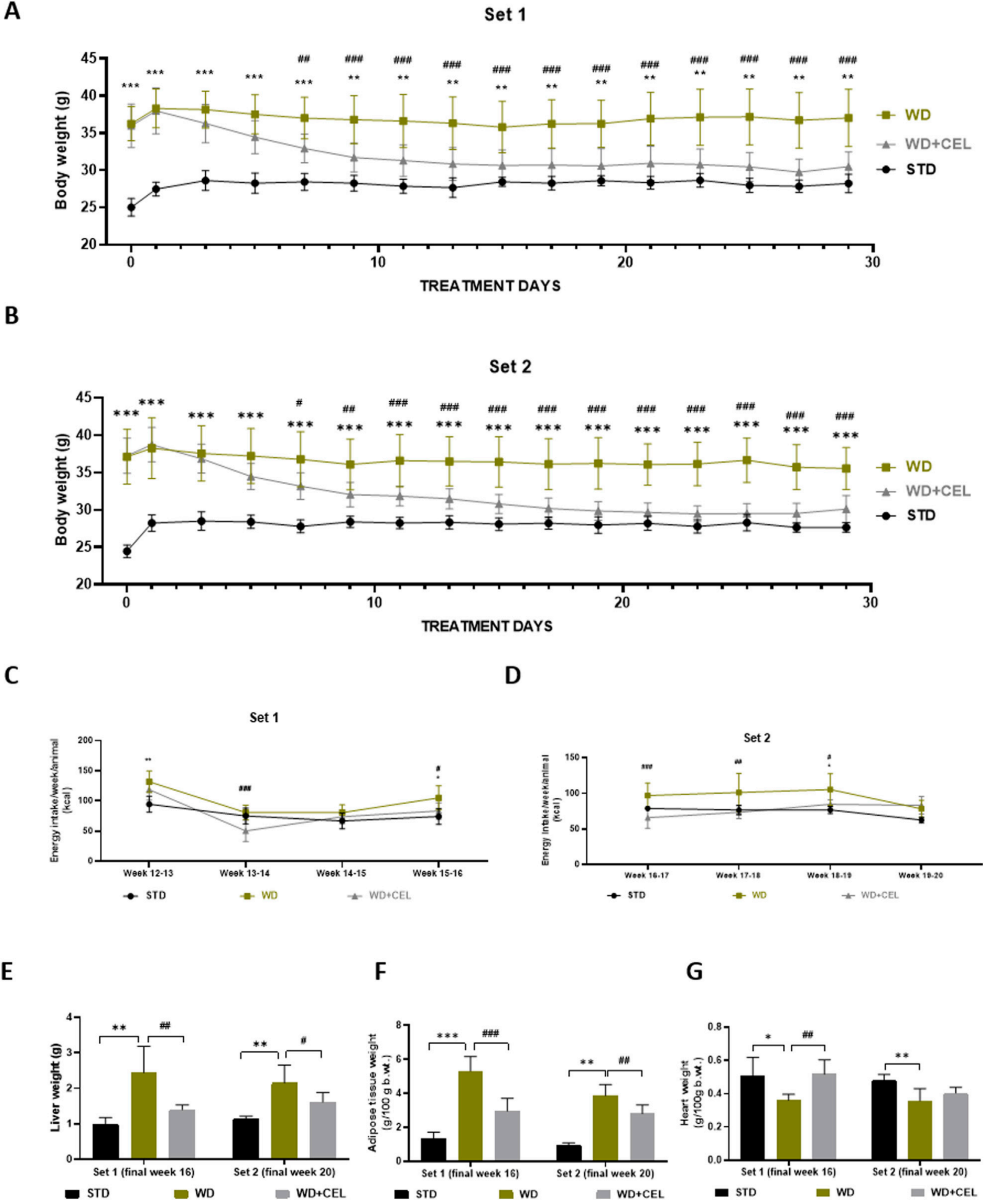
Effect of 4-week celastrol (CEL) treatment on mouse body and liver weight, energy consumption, and adipose tissue-to-body and heart-to-body weight ratios in each set of *in vivo* experiments. **(A)** Each second-day body weights of the experimental set 1 mice during weeks 12–16 (note that the first measurement was taken after 12-h fasting). **(B)** Each second-day body weights of the experimental set 2 mice during weeks 16–20 (note that the first mouse weighing was done after 12-h fasting). **(C)** Final weekly energy consumption per mice of set 1 (measured during weeks 12–16). **(D)** Final weekly energy consumption per mouse of set 2 (measured during weeks 16–20). **(E)** Liver weight. **(F)** Ratio of abdominal plus epididymal fat weight to total body weight. **(G)** Heart-to-body weight ratio. Data are expressed as means ± SD (n = 3 for negative controls, n = 7–8 for positive controls and CEL treatment), where **p < 0.01, ***p < 0.001, and ****p < 0.001 when comparing respective positive control (WD group) against negative control (STD group) and ^#^p < 0.05, ^##^p < 0.01, ^###^p < 0.001, and ^####^p < 0.001 when comparing CEL treatment (WD+CEL group) against positive control, as assessed by one-way **(E–H)** or two-way **(A–D)** ANOVA with the *post hoc* Bonferroni test.

We successfully adopted WD/FG-induced obesity and MASLD/MASH in C57BL6J male mice (Arora et al., 2023), as evidenced by the progressive increase in body weights (Supplementary Figure S1), liver weight, and fat-to-body weight ratio in the positive control groups of both sets of experiments (Figure 2). CEL treatment (i.e., CEL + WD group) significantly decreased mouse body weights after 1 week of treatment when compared to the body weights of the positive control (i.e., WD group) in both sets of experiments. This weight loss persisted until the end of the experiment (Figures 2A, B). CEL significantly reduced food consumption and energy intake compared to positive controls, which was more pronounced during the first 2 weeks of the treatment (Supplementary Figures S2; Figures 2C, D, respectively). CEL significantly decreased the liver weight in both sets of experiments (Figure 2E). The absolute amount of white intra-abdominal plus epididymal fat tissue and fat-to-body weight ratios was also significantly decreased by CEL throughout the study (Figure 2F). As the absolute heart weights were not modified throughout the groups and sets, the heart-to-body weight ratio was significantly decreased in both positive controls and completely restored by CEL during set 1 (Figure 2G).

### 3.2 The effect of celastrol treatment on mouse glycemia and serum liver and kidney biochemistry markers

**FIGURE 3:**
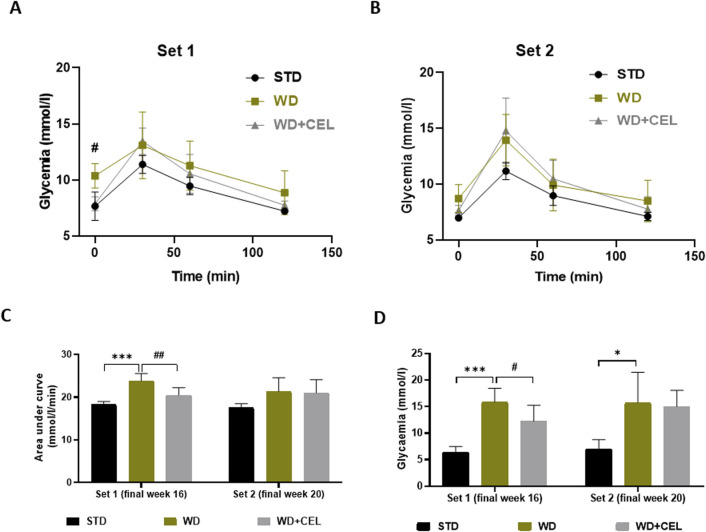
Effect of celastrol (CEL) on serum glucose levels in each experimental set of WD/FG-induced obesity and MASLD/MASH in mice. **(A)** Oral glucose tolerance test (OGTT) glucose levels at week 16 of set 1. **(B)** OGTT glucose levels at week 20 of set 2. **(C)** Area under the curve of OGTT glycemia calculated using the trapezoidal rule. **(D)** Terminal 12-h fasting serum glucose levels. Data are expressed as means ± SD (n = 3 and 7–8/group as noted in Table 1), where *p < 0.05, **p < 0.01, and ***p < 0.001 compared to the respective positive control (WD group) against negative control (STD group) and ^#^p < 0.05 and ^##^p < 0.01 compared to CEL treatment (WD+CEL group) against positive control, as assessed by one-way **(C, D)** or two-way **(A, B)** ANOVA with the *post hoc* Bonferroni test.

At the end of experimental set 1 (i.e., week 16), mice fed WD/FG showed an elevated overall OGTT curve, as evidenced by the significantly increased glycemic AUC that was substantially reduced by CEL (Figures 3A, C). The same significance was also detected for 12-hour fasting glucose levels (Figure 3D). At the end of experimental set 2 (i.e., week 20), only 12-hour fasting glycemia was remarkably elevated in positive controls (Figures 3B, D).

**FIGURE 4:**
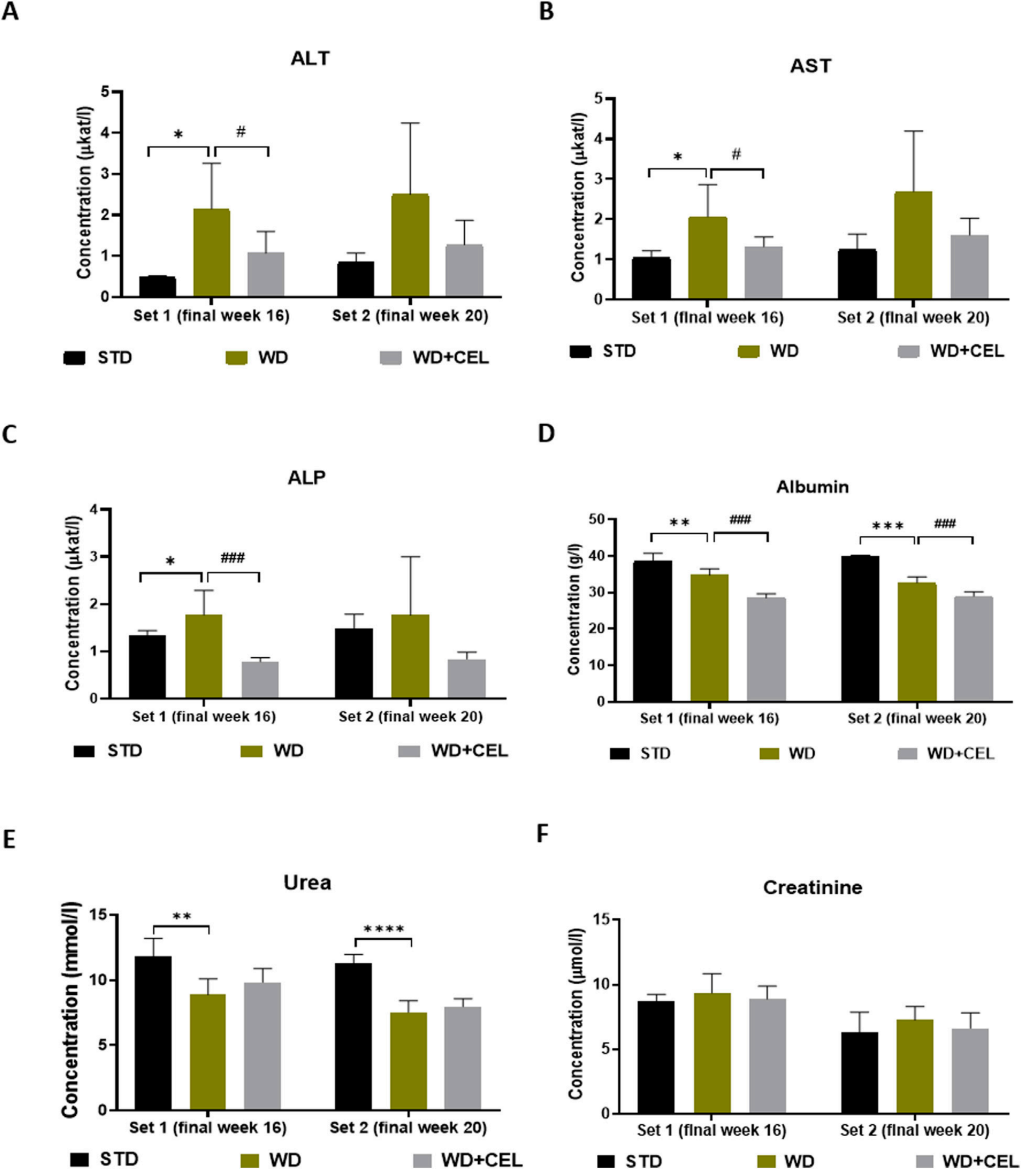
Effect of celastrol (CEL) treatment on biochemical serum markers of liver and kidney functions in each experimental set of WD/FG-induced obesity and MASLD/MASH mouse model. **(A)** serum ALT, **(B)** serum AST, and **(C)** serum ALP catalytic activity concentrations, **(C)** serum ALP levels, **(D)** serum albumin levels, **(E)** serum urea levels, and **(F)** serum creatinine levels. Data are expressed as means + SD (n = 3 and 7–8/group, as noted in Table 1), where *p < 0.05, **p < 0.01, ***p < 0.001, and ****p < 0.001 compared to the positive control (WD group) against the respective negative control (STD group) and ^###^p < 0.001 compared to CEL treatment (WD+CEL group) against the positive control, as assessed by one-way ANOVA with the *post hoc* Bonferroni test.

CEL treatment significantly reduced or displayed a tendency to drop (not statistically significant, n.s.) all liver enzyme activities at week 16 or week 20 (Figures 4A–C). Serum albumin comprises an essential endogenous protein synthesized by the liver. Moreover, the decrease in the serum albumin concentration is suggested to be an essential clinical predictor for MASLD-associated hepatic damage (Kawaguchi, K. et al., 2021). In our study, WD/FG-induction displayed a progressive significant decrease in serum albumin levels, which were highly significantly further decreased by the CEL treatment (Figure 4D). Additional decline could be caused by the very high affinity of CEL to serum albumin, which can interfere with the colorimetric assay method (especially with using bromocresol green, as in our case) to estimate the albumin concentration (Zhang et al., 2009; Fan et al., 2022). A significant reduction of serum urea was also seen in all positive controls against negative controls, probably due to decreased liver synthesis (Arora et al., 2023). Serum creatinine levels decreased with time (e.g., animal age) but were not affected by WD/FG. The CEL treatment did not produce any further alterations in serum urea and creatinine levels at any time point, indicating its safety for the kidney (Figures 4E, F).

### 3.3 The effect of celastrol treatment on serum lipids, liver triglyceride content, oxidative stress markers, and TNF-alpha

**FIGURE 5:**
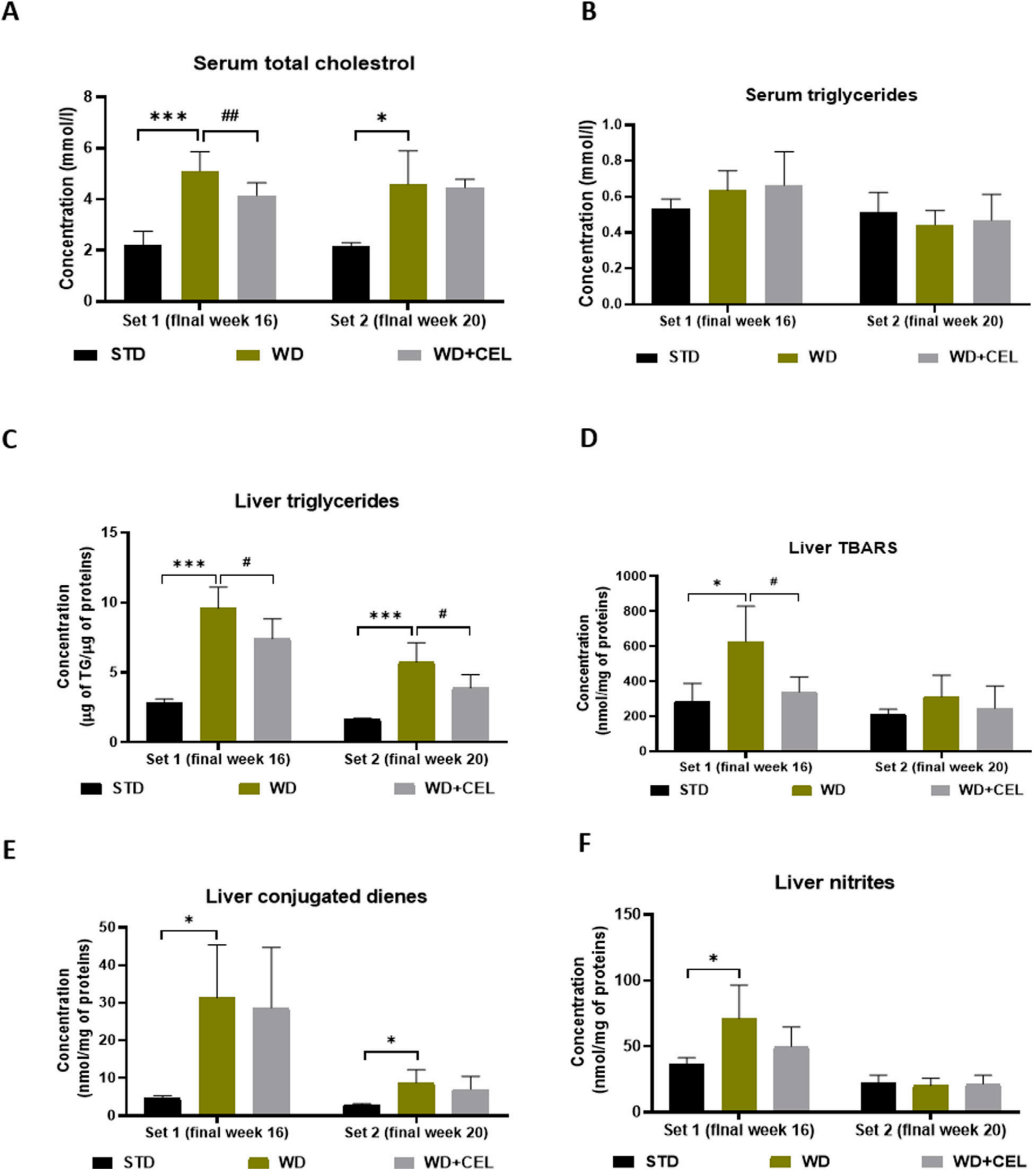
Effect of celastrol (CEL) treatment on concentrations of mouse serum total cholesterol **(A)**, serum triglycerides **(B)**, liver triglyceride (TG) content **(C)**, liver TBARS **(D)**, liver conjugated dienes **(E)**, and liver nitrites **(F)** in both sets of the *in vivo* experiment. Data are expressed as means + SD (n = 3 and 7–8/group, as noted in Table 1), *p < 0.05 and ***p < 0.001 compared to the respective positive control (WD group) against the negative control (STD group) and ^#^p < 0.05 and ^##^p < 0.01 compared to CEL treatment (WD + CEL group) against the positive control, as assessed by one-way ANOVA with the *post hoc* Bonferroni test.

In both sets of *in vivo* experiments, the atherogenic WD/FG diet significantly enhanced the concentrations of serum total cholesterol when compared to negative controls. CEL treatment decreased this serum lipid marker significantly only at the end of set 1 (Figure 5A). Although serum triglyceride levels were not affected by either WD/FG or WD/FG + CEL (Figure 5B), the liver TG content was highly significantly increased in positive controls and, conversely, remarkably (p < 0.05) reduced after CEL treatment in both sets (Figure 5C).

Animals in the positive control (i.e., WD) group displayed significantly enhanced concentrations of liver TBARS, conjugated dienes, nitrites, and TNF-α at the end of week 16 (i.e., set 1) when CEL treatment was able to significantly reduce only TBARS. At the end of week 20 (i.e., set 2), WD/FG alone significantly increased only the conjugated diene content in the liver, while CEL had no additional effect on any of the liver oxidative stress markers and TNF-α (Figures 5D–F; Supplementary Figure S3, respectively).

### 3.4 The effect of celastrol treatment on the liver morphology of diet-induced MASLD/MASH

**FIGURE 6:**
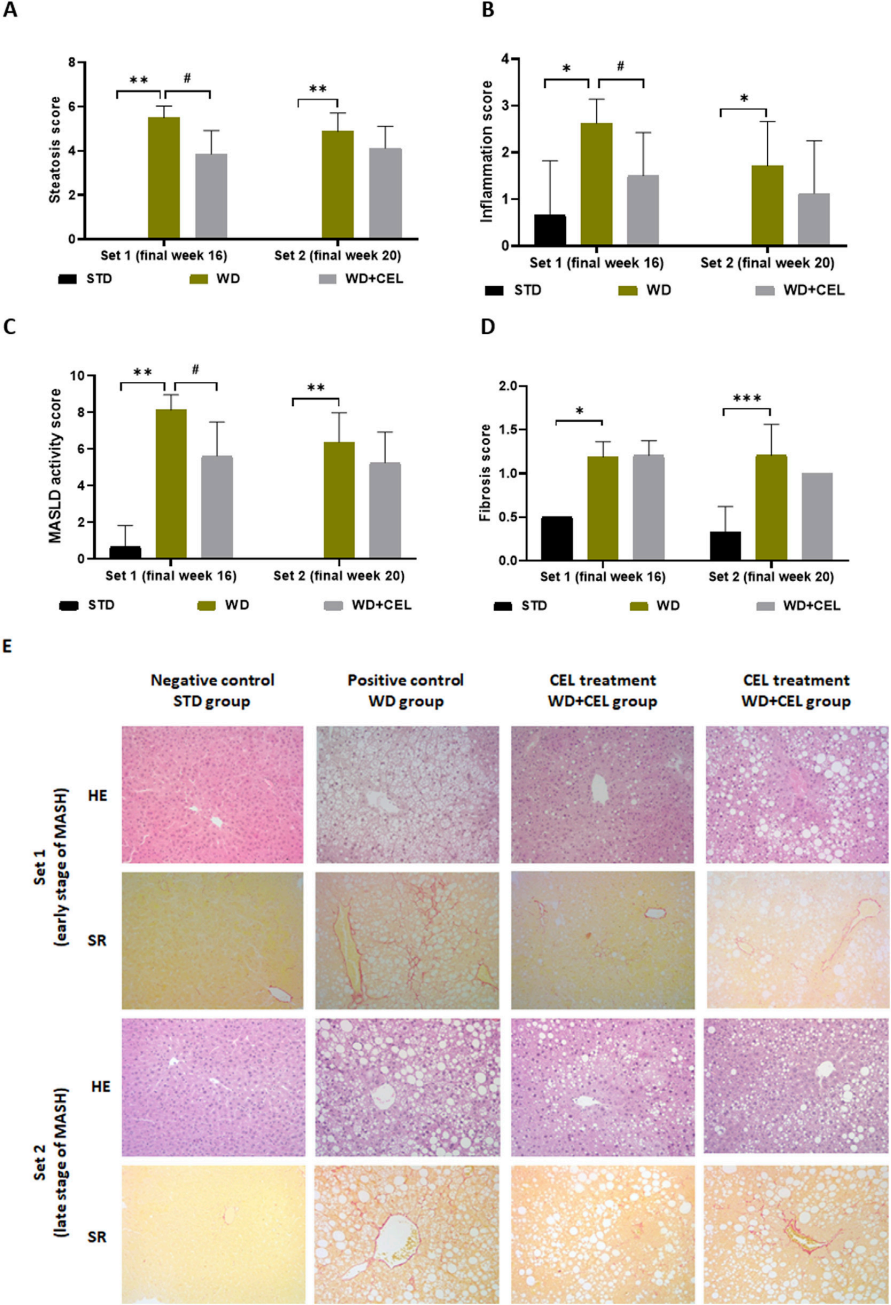
Impact of celastrol treatment on mouse MASLD/MASH histopathological scoring and staging of the experimental set 1 and set 2 (representing the end of weeks 16 and 20, respectively), including **(A)** total cell macrovesicular, microvesicular, and hypertrophy liver steatosis score, **(B)** total liver inflammation score, **(C)** MASLD activity score, and **(D)** liver fibrosis score. The data are expressed as the means + SD, where *p < 0.05, ***p < 0.01, and **p < 0.01 when comparing the positive control (WD group, n = 7–8) to the respective negative control (STD group, n = 3) and ^#^p < 0.05 when comparing CEL treatment (WD+CEL, n = 8) to the positive control, as assessed by the Kruskal–Wallis test with *post hoc* Dunn’s multiple comparison test. Note: some data are missing for negative controls as the scoring values were zero for all three samples. Similarly, some SDs are missing as the data were completely equal for all livers in the respective group, that is, the SDs were zero. **(E)** Example images of liver sections used for histopathological evaluation after staining with hematoxylin–eosin (HE) or Sirius red (SR) at a magnification of ×200.

Macroscopically, there was a noticeable difference between the negative and positive control livers, which were hypertrophied and much lighter due to fat accumulation (Supplementary Figure S4). The positive controls displayed highly significantly elevated total steatosis, inflammation, MASLD activity, and fibrosis scores in mouse livers of both sets, confirming MASH. However, the overall fibrosis caused in mouse fed on WD/FG was low and reached only that of stage 1B in average (i.e., 1.67), representing moderate perisinusoidal fibrosis at the microscopic level. At the end of week 16, CEL significantly reduced liver steatosis, inflammation, and the total MASLD activity score and ameliorated liver morphology. CEL treatment displayed a similar pattern of slight reduction (n.s.) in all histological scores and overall liver morphology at the end of week 20 (Figure 6), when the liver became harder with the disorganization of typical microarchitecture, prevailing macrovesicular steatosis and increasing the number of fibroblast-like cells. Moreover, the development of liver tumors was noted in one positive control case of set 2 (Supplementary Figure S4).

### 3.5 The effect of celastrol treatment on MASLD/MASH-related liver gene expression in diet-induced obesity of mice

**FIGURE 7:**
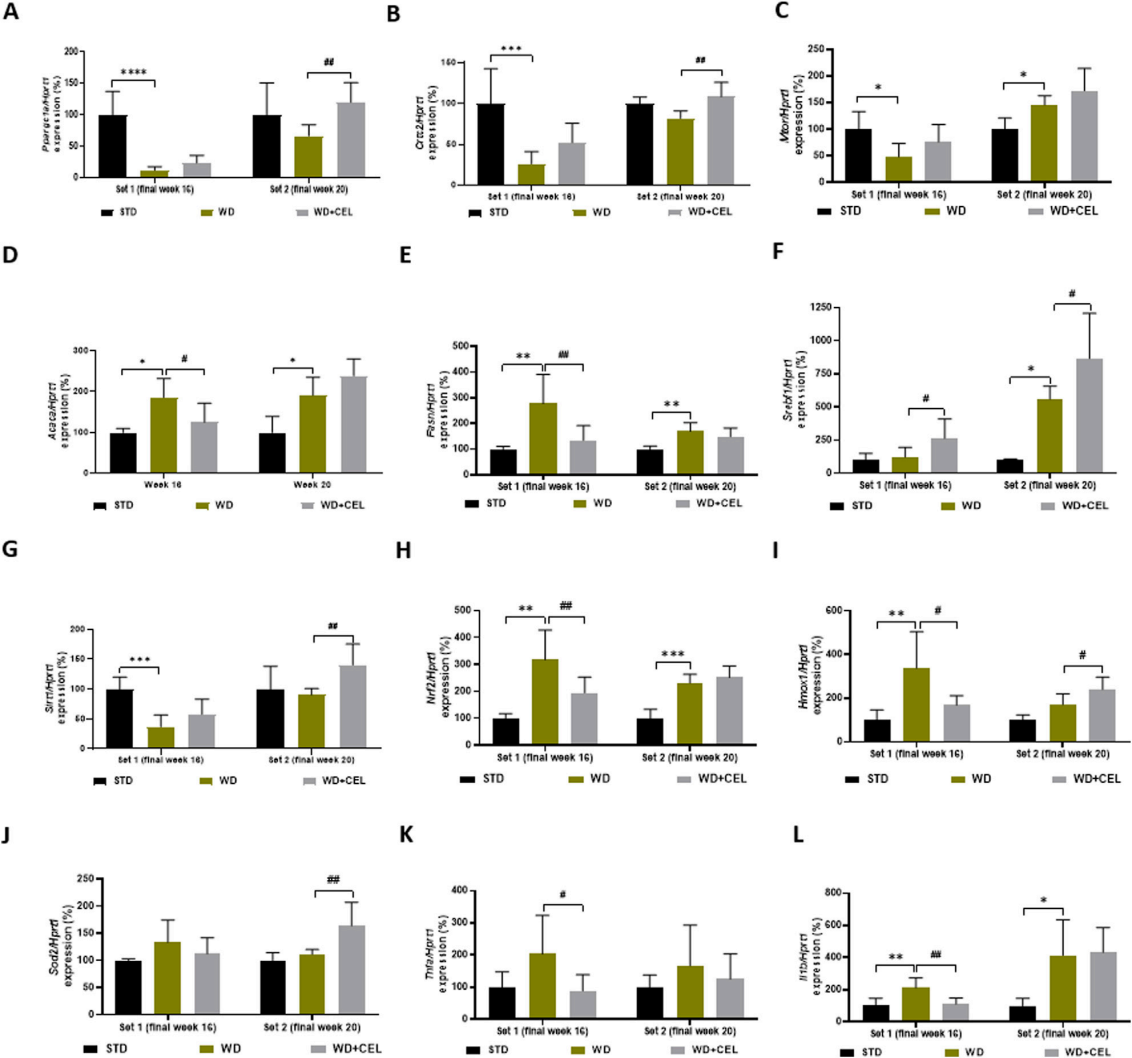
Effect of celastrol treatment on liver expression of selected genes evaluated in each experimental set of WD/FG-induced obesity and the MASLD/MASH mouse model, where **(A)** *Ppargc1a* (peroxisome proliferative activated receptor gamma, coactivator 1 alpha), **(B)** *Crtc2* (CREB, cAMP response element-binding protein, regulated transcription coactivator 2), **(C)** *Mtor* (mechanistic target of rapamycin kinase), **(D)** Acaca (acetyl-coenzyme A carboxylase alpha), **(E)** *Fasn* (fatty acid synthase), **(F)** *Srebf1* (sterol regulatory element-binding transcription factor 1), **(G)** *Sirt1* (sirtuin 1), **(H)** *Nrf2* (also Nfe2l2, nuclear factor, erythroid derived 2, like 2), **(I)** *Hmox1* (heme oxygenase 1), **(J)** *Sod2* (superoxide dismutase 2, mitochondrial), **(K)** *Tnfa* (tumor necrosis factor alpha), and **(L)** *Il1b* (interleukin 1 beta) represent specific gene expressions. Data are expressed as means + SD (in percentage of average negative control ∆∆Ct value calculated against the expression of housekeeping gene *Hprt1*, hypoxanthine phosphoribosyltransferase 1), where *p < 0.05, **p < 0.01, ***p < 0.001, and ****p < 0.0001 compared to the respective positive control (WD group, n = 6–8) against the negative control (STD group, n = 3) and ^#^p < 0.05 and ^##^p < 0.01 when comparing CEL treatment (WD + CEL, n = 7–8) against the positive control, as assessed by one-way ANOVA.

Some genes illustrated variation during the progression from steatosis to hepatitis at different time points, while other genes indicated the same pattern in both sets. The expression of the gene *Ppargc1a*, which encodes a transcriptional coactivator PGC-1α affecting energy metabolism, was downregulated by WD/FG at both sets; however, it was significant only at the end of week 16. On the other hand, CEL significantly upregulated *Ppargc1a* gene expression only at the end of week 20 (Figure 7A). Similar trends of the atherogenic diet and CEL were observed for the *Crtc2* gene-encoding CRTC2 protein, which is also involved in glucose metabolism, lipogenesis, and other various cell processes (Figure 7B). As CRTC2 can regulate mTOR signaling pathway transduction (Zheng et al., 2023), we also screened for *Mtor* gene expression. Similar to *Crtc2* at week 16, *Mtor* gene expression was significantly downregulated by WD/FG. On the other hand, it was significantly upregulated by WD/FG at week 20, which was not affected by CEL (Figure 7C).

Gene expression levels of *Acaca* and *Fasn* that code for important lipogenic enzymes promoting *de novo* lipogenesis and adipogenesis were induced in mouse livers of positive controls of both sets. However, CEL-treatment normalized *Acaca* and *Fasn* mRNA levels only at the end of week 16. At week 20, CEL further enhanced upregulated *Acaca* gene expression (Figures 7D, E). Surprisingly, unlike *Acaca* and *Fasn*, the expression of the *Srebf1* gene was significantly increased only in set 2, and CEL significantly increased its expression in both experimental sets (Figure 7F).

Expression of genes involved in cell survival, senescence, and/or oxidative status (i.e., decreasing oxidative stress), such as *Sirt1*, *Nrf2*, *Hmox*, and *Sod2*, produced highly variable results. For example, *Sirt1* was significantly downregulated by an atherogenic diet at week 16 and remarkably upregulated by CEL at week 20 (Figure 7G). Although *Sirt1* should play a key role in activating the Nrf2/ARE (antioxidant response element) signaling pathway and protecting against oxidative stress (Zhuang et al., 2021), the *Nrf2* gene was highly significantly upregulated by WD/FG throughout the experiment; however, CEL decreased its mRNA levels remarkably only during set 1 (Figure 7H). Nrf2, a crucial transcription factor, plays a pivotal role in determining the liver’s antioxidant capacity and detoxification status (Shin et al., 2013). Nrf2, among others, coordinates gene expression of *Hmox1*, which was affected in the same way as *Nrf2* only at the end of week 16. Later on, *Hmox1* mRNA was further increased by CEL (Figure 7I). Only at the end of week 20, CEL also very significantly increased the otherwise unaffected expression of the *Sod2* gene, which codes the critical antioxidant enzyme SOD-2 (Figure 7J).

For the evaluation of the inflammatory pathway, we assessed the liver expression of genes coding TNF-α and IL-1β cytokines. In contrast to liver TNF-α protein levels (Supplementary Figure S3), *Tnfa* mRNA levels were not affected by WD/FG; however, it was significantly downregulated by CEL only at week 16 (Figure 7K). Gene expression of *Il1b* was significantly upregulated by the atherogenic diet during both sets, and CEL treatment significantly reversed it only at the end of set 1 (Figure 7L).

### 3.6 The effect of celastrol treatment on the gene expression of galaninergic system members in the liver and heart ventricle tissues of Western diet-induced obese mice

We realized that the quantitative gene expression of the members of the galanin family in mouse heart ventricles was the strongest for *Galr2*, followed, in a descending manner, by *Gal*, *Galr1*, and *Galr3*. *Galp* gene expression was very low and under or at the limit of detectability in all heart ventricle samples; therefore, it was not possible to meaningfully analyze this. In mouse liver tissue, only *Galr2* gene expression could be quantitatively evaluated because the expression of other genes (i.e., *Gal*, *Galr1*, and *Galr3*) was very low.

**FIGURE 8:**
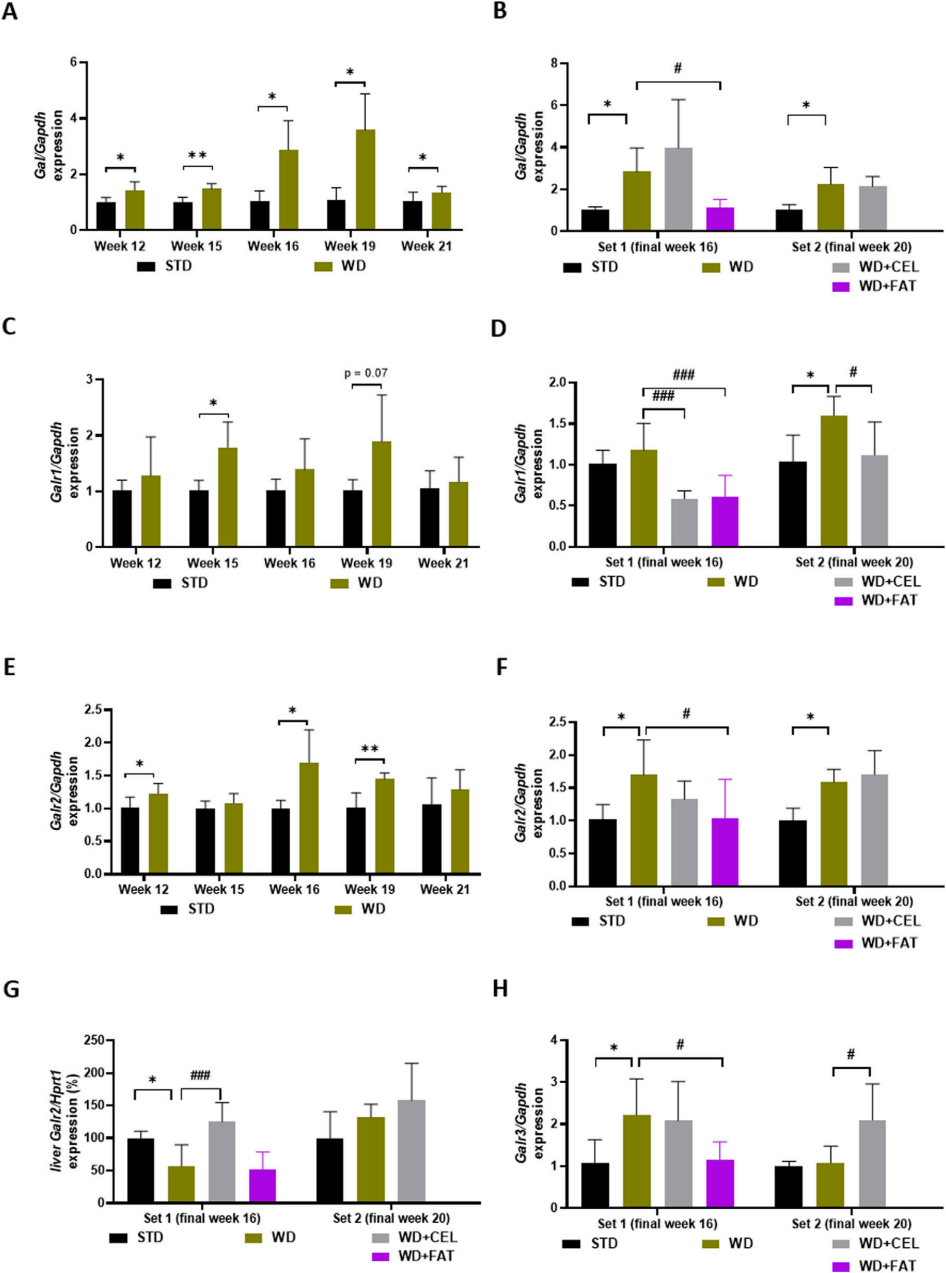
Effect of celastrol (CEL) and fatostatin (FAT) treatment on gene expression of members of the galanin system in mouse heart ventricles **(A–F, H)** and livers **(G)** in WD/FG-induced obesity and MASLD/MASH model, where **(A, B)** *Gal* (galanin gene), **(C, D)** *Galr1* (galanin receptor 1 gene), **(E–G)** *Galr2* (galanin receptor 2 gene), and **(H)** *Galr3* (galanin receptor 3 gene) represent the specific gene expression of the exploratory pilot study **(A, C, E)** and the *in vivo* experiments covering set 1 and set 2 lasting for 16 and 20 weeks, respectively **(B, D, F–H)**. Data are expressed as means + SD (as average of negative control ∆∆Ct value calculated against the expression of housekeeping gene), where *p < 0.05, **p < 0.01, and ***p < 0.001 when comparing the respective positive control (WD group, n = 4 for hearts sampled at weeks 15 and 19, n = 6 for hearts sampled at weeks 12 and 16, n = 7 for livers from set 1, and n = 8 for others) against the respective negative control (STD group, n = 3 or n = 5 for only hearts sampled at week 21) and ^#^p < 0.05 and ^###^p < 0.001 when comparing CEL or FAT treatment (WD+CEL or WD+FAT, n = 6 for hearts only, n = 8 for livers of both sets) against the respective positive control, as assessed by Student’s t-test [for graphs **(A, C, E)**] or one-way ANOVA [for graphs **(B, D, F–H)**].

As for specific qRT-PCR results in mouse heart ventricles, there was a highly detectable expression of the *Gal* gene increasing with age in negative controls, which was further significantly upregulated by the Western-type diet, with the peak expression at weeks 16–19 (Figure 8A). CEL had no additional effect, while FAT significantly downregulated *Gal* expression (Figure 8B). The expression of the *Galr1* gene was lower than that of *Gal*; however, it exerted a similar pattern (Figure 8C). CEL and FAT addition to WG/FG highly significantly decreased the *Galr1* gene expression, which was slightly increased (n.s.) or significantly upregulated by the Western-type diet at the end of weeks 16 and 20, respectively (Figure 8D). In the heart ventricles, there was very high expression of the *Galr2* gene, which was relatively stable, concerning the mouse age and significantly upregulated by WD/FG, with the peak expression at weeks 16–20. CEL had no additional effect on this, while FAT significantly downregulated enhanced *Galr2* mRNA levels at the end of week 16 (Figures 8E, F). Interestingly, *Galr2* gene expression in the mouse liver of set 1 was affected in a completely different manner: it was significantly downregulated by WD/FG, highly significantly increased by CEL, and unchanged by FAT when compared to that in positive controls (Figure 8G). Finally, there was a very low expression of the *Galr3* gene, generally in the heart, which was nearly undetectable until week 15, with a peak at week 19 and a remarkable drop at week 21 in both the negative and positive controls. At the end of week 16, *Galr3* mRNA levels were borderline detectable and significantly enhanced by WD/FG in heart ventricles, which remained unaffected by CEL; however, they were significantly downregulated by FAT treatment. At the end of week 20, *GalrR3* gene expression was significantly upregulated by the addition of CEL to the Western-type diet, of which *Galr3* mRNA levels were the same as for the negative control group (Figure 8H).

### 3.7 The effect of celastrol treatment on the MASLD/MASH-related liver protein expression of SREBP1 and GalR2

**FIGURE 9:**
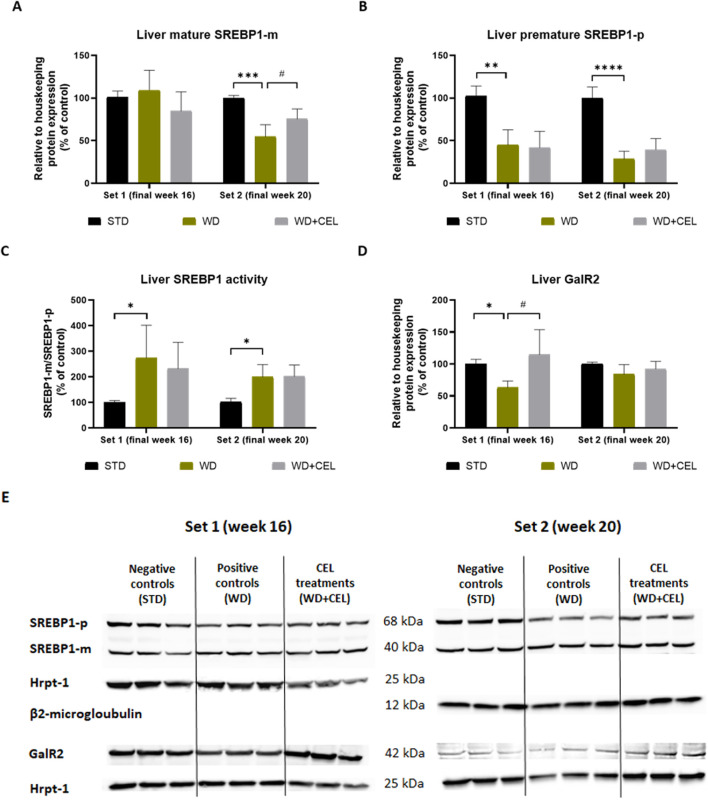
Effect of celastrol (CEL) treatment on liver protein expression of mature SREBP1 (SREBP1-m), SREBP1 precursor (SREBP1-p), and galaninergic receptor type 2 (GalR2) in WD/FG-induced obesity and the MASLD/MASH model of the *in vivo* experiments covering set 1 and set 2 lasting for 16 and 20 weeks, respectively. **(A)** Quantification of SREBP-m protein expression. **(B)** Quantification of SREBP-p protein expression. **(C)** SREBP1 activity calculated as the ratio of SREBP1-m/SREPBP1-p relative protein expressions. **(D)** Quantification of GalR2 protein expression. **(E)** Example of Western blot analyses of SREBP-p, SERBP-m, GalR2, Hrpt-1, and beta2-microglobulin proteins. Data in the graphs are expressed as means + SD (as an average of negative control value calculated against the expression of housekeeping protein), where *p < 0.05, **p < 0.01, ***p < 0.001, and ****p < 0.0001 when comparing the respective positive control (WD group, n = 5 and n = 6 for set 1 and set 2, respectively) against the respective negative control (STD group, n = 3) and ^#^p < 0.05 when comparing CEL treatment (WD + CEL group, n = 5 and n = 6 for set 1 and set 2, respectively) against the respective positive control, as assessed by one-way ANOVA.

In liver homogenates, we detected two Western blot lines of positive bands (Figure 9A), which represent the immature and mature forms of SREBP1 (Tiscione et al., 2019). Interestingly, the protein expression of mature (cleaved, active, and nuclear) SREBP1-m was not affected in set 1; however, it significantly decreased in set 2 of the *in vivo* experiment and returned toward negative control levels under CEL treatment (Figure 9A). The amount of its precursor (immature and uncleaved) SREBP1-p highly significantly decreased both in positive and CEL-treated groups (Figure 9B), suggesting SREBP1-p consumption due to its activation through the cleavage in livers of mice on the WD/FG diet. Therefore, we calculated the SREBP1 activity as the ratio of SREBP1-m/SREPBP1-p (relative protein expressions), which was significantly increased in positive controls in both sets. CEL had no additional effect on this SREBP1 activity (Figure 9C).

In accordance with *Galr2* mRNA expression, CEL significantly upregulated GalR2 protein expression, which was apparently reduced in positive control mouse livers of only experimental set 1 (Figure 9D).

### 3.8 The immunohistochemical detection of GalR1 in the heart tissue

**FIGURE 10:**
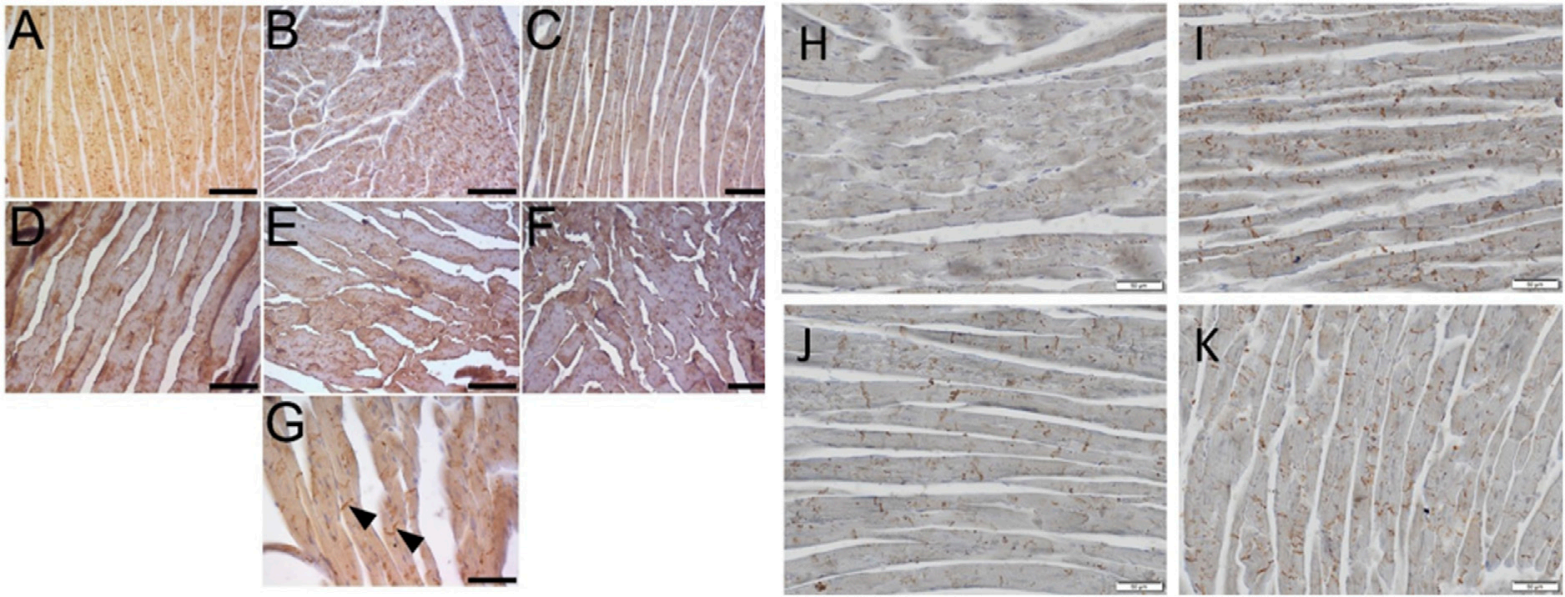
Immunohistochemical detection of GalR1 in hearts of mice on standard diet (STD, negative controls) and Western-type diet (WD, positive controls) for overall 16 or 20 weeks and mice on WD treated for the last 4 weeks with celastrol (CEL) or fatostatin (FAT). Example images taken by a Leica DMLB microscope equipped with a Leica MC170 HD camera show the results of immunoperoxidase reaction to detect GalR1 in the heart sections. All images show the left ventricular wall, where **(A–C)** represent myocardia after 20 weeks (corresponding to set 2), and **(D–K)** represent myocardia after 16 weeks (corresponding to set 1) of *in vivo* experiments. Mouse hearts of STD **(A, D, G, H)**, WD **(B, E, I)**, WD + CEL **(C, F, J)**, or WD + FAT **(K)** groups. GalR1 immunoreactivity is visible in cardiomyocytes as a diffuse reaction product in the cytoplasm **(A–F)** and a concentrated reaction product in intercalated discs of cardiomyocytes **(G–K)**, best seen in transversal sections (arrows) **(G)** and at higher magnification **(H–K)**. Scale bar in **(A–F)** = 200 μm, in **(G)** = 100 μm, and **(H–K)** = 50 µm.

We performed advanced immunohistochemical detection of GalR1 in the hearts of mice on STD, WD/FD, and mice on the Western-type diet treated with CEL or FAT in two individual experimental sets. GalR1 was detected in all samples, both in the atria and in the ventricles. The intensity of the immunohistochemical reaction was similar in the hearts of mice from two different sets and different experimental groups (Figures 10A–F); however, there was considerably higher immunoreactivity in positive controls and lower immunoreactivity in heart sections of CEL- and FAT-treated mice (Figures 10H–K). The reaction product was mostly diffusely distributed in the cytoplasm of cardiomyocytes. We often found a prominent reactivity at the intercalated discs (Figure 10G). This staining pattern was not characteristic for any of the groups. Due to the variable GalR1 positivity, which is diffused in the cytoplasm compared to the concentrated one in the intercalary discs, it was not possible to perform a relevant quantitative analysis.

### 3.9 The effect of celastrol and M871 on palmitic-induced hepatocyte lipotoxicity *in vitro*

Pre-treatment of primary hepatocytes with CEL at a nontoxic concentration (500 nM) had no effect on palmitic acid (PA)-induced lipotoxicity, as evidenced by cell viability, ALT release, and Red Oil O stain accumulation (for *in vitro* methods and Supplementary Figure S5, see Supplementary Material). In combination with PA and/or M871, CEL exhibited a noteworthy reduction in nitrite production by hepatocytes (Supplementary Figure S5C). The GalR2 inhibitor, an M871 compound (100 nM), significantly increased PA-induced cytotoxicity and nitrite levels (as an oxidative stress indicator). On the other hand, M871 rather decreased lipid accumulation in hepatocytes (Supplementary Figures S5D–F).

## 4 Discussion

At the beginning of the Discussion section, it is important to stress that the aim of the presented study was not to prove or develop a mouse MASLD/MASH model, as we already did this in our previous study (Arora et al., 2023). Therefore, and to reduce the number of mice to the minimum, we used only three mice in the negative controls (STD group) as a type of background for the whole experiment, which caused some results in the positive control (WD group) not reaching statistically significant values. This may appear to be the main shortcoming of our study. Another shortcoming of our study is the fact that the impact of CEL has been exclusively studied only in male subjects, although MASLD prevalence is increasing in women with polycystic ovary syndrome and estrogen deficiency, especially after menopause, leading to higher risks of MASH and advanced fibrosis compared to men of the same age (Eng et al., 2023). However, as our main aim was to evaluate the effect of celastrol treatment (WD + CEL group) against WD/FG-induced obesity and MASLD/MASH, we preferably statistically analyzed the differences between WD and WD+CEL groups of male mice, which we discuss in the following text.

In the current study, we originally demonstrated that celastrol has beneficial effects on the Western-type diet-induced obesity and progression of MASLD to MASH in mice via reduced liver inflammation, enhanced antioxidant defense, and improved lipid and glucose metabolism in at least one set of *in vivo* experiments. Other studies documented similar ameliorative effects of CEL on HFD-induced obesity and fatty liver in mice (Li M. et al., 2022; Zhang et al., 2016; Ye et al., 2024). The body weight-lowering effect of CEL in our study could be at least partially explained by decreased food and energy intake. It can be interpreted by the suppression of blood GAL and GALP levels and Gal/GalR1/GalR3, NPY, and leptin expressions in the hypothalamus of obese mice, where CEL works as a leptin sensitizer (Fang et al., 2019; Liu et al., 2015; Marcos and Coveñas, 2021; Takenoya et al., 2018; Ye et al., 2024). However, we have shown that the body, liver, and fat tissue weights of CEL-treated mice on the diet were significantly lower at the end of the experimental sets, although the reduced energy end/or food intake already did not differ from that of positive controls that also had considerably reduced WD consumption, namely, in set 2. Potentially, central (hypothalamic) resistance to the long-term effect of celastrol could also develop as the development of tissue galanin resistance with persistent galanin levels in obese subjects has been described before (Marcos and Coveñas, 2021). Therefore, factors other than central anorexigenic effects could also contribute to CEL complex ameliorative properties on the body, liver, and fat tissue weights and energy metabolism (i.e., improved glucose tolerance and reduced fasting glucose levels, reduced serum total cholesterol, and decreased liver triglyceride content).

To understand the progression of metabolic disease represented by MASLD/MASH, it is crucial to assess the expression of central metabolic, oxidative status, and inflammatory genes through time. This assessment provides valuable insights into the complex impact of celastrol treatment at various stages of MASH pathogenesis in the WD/FG-induced obesity experimental model. Therefore, mRNA levels (i.e., gene expression) of the selected genes involved in MASLD/MASH progression were determined in mouse livers by employing quantitative RT-PCR: *Ppargc1a* (peroxisome proliferative activated receptor, gamma, coactivator 1 alpha), *Crtc2* (CREB, cAMP response element-binding protein, regulated transcription coactivator 2), *Mtor* (mechanistic target of rapamycin kinase), *Fasn* (fatty acid synthase), *Acaca* (acetyl-coenzyme A carboxylase alpha), *Srebf1* (sterol regulatory element-binding transcription factor 1), *Nrf2* (also Nfe2l2, nuclear factor, erythroid derived 2, like 2), *Hmox1* (heme oxygenase 1), *Sirt1* (sirtuin 1), *Sod2* (superoxide dismutase 2, mitochondrial), *Tnfa* (tumor necrosis factor alpha), and *Il1b* (interleukin 1 beta). CEL produced metabolic benefits through the activation of PGC-1α, leading to enhanced energy expenditure and browning of white adipose tissue by regulating mitochondrial function and biogenesis (Fang et al., 2019; Li M. et al., 2022). Throughout our *in vivo* experiment, CEL upregulated *Ppargc1a* gene expression that was suppressed by WD/FG, resembling liver-specific-deficient PGC-1α mice with manifested hepatic steatosis (Rodgers et al., 2005). We have observed similar patterns in *Crtc2* and *Sirt1* gene regulation by the Western-type diet and CEL. CRTC2 is a transcriptional coactivator of CREB (cAMP response element-binding protein), which plays a role in glucose, lipid, and energy metabolism through increased PGC-1α transcription and regulation of several pathways, including the cAMP and mTOR pathways, and SRBEP1/2 (Zheng et al., 2023). There are no specific studies on *Crtc2* gene expression in response to CEL, while celastrol’s impact on SIRT1 expression was described to contribute to its protective effects against fatty liver (Zhang et al., 2016). SIRT1 deactivated CRTC2 and SREBP-1c, decreasing early gluconeogenesis and lipogenesis, and activated PGC-1α, reducing adiposity and lipogenesis while promoting fatty acid oxidation (Elibol and Kilic, 2018). Reciprocally, CREB and PGC-1α can upregulate SIRT1 (Molla et al., 2020), indicating that PGC-1α, CRTC2, and SIRT1 are interconnected players in metabolic regulation, impacting processes like gluconeogenesis and lipid metabolism. Therefore, we can assume that the hepatoprotective and beneficial metabolic effects of CEL in our study can, to some extent, be mediated by the CRTC2-PGC-1α-SIRT1 pathway. Furthermore, WD/FG increased serum total cholesterol, adipose tissue weight, liver triglyceride content, and steatosis score that were accompanied by significant upregulation of lipogenic genes like *Acaca* and *Fasn*. CEL significantly decreased all these lipid metabolism parameters and both genes in the set 1 experiment, which is in accordance with other animal studies (Hu et al., 2018; Zhang et al., 2016). Later, as MASH progressed, the effect of CEL was not as evident and *Acaca* gene expression even increased. *Fasn* and *Acaca* are the target genes for sterol regulatory element-binding protein 1 (SREBP1) transcription factor, which is involved in glucose metabolism; fatty acid, cholesterol, and TG synthesis; and adipogenesis and is encoded by the *Srebf1* gene (Soyal et al., 2015). CEL can also target SIRT1 to promote AMPK-α phosphorylation and inhibit Srebp-1c-mediated lipid synthesis against oxidative stress and inflammation (Li M. et al., 2022). Unpredictably, CEL significantly upregulated increased *Srebf1* mRNA in our *in vivo* experiments. We assumed that this was a compensatory mechanism when CEL might inhibit the activation of SREBP1 protein through the induction of the CRTC2-PGC-1α-SIRT1 pathway as CRTC2 modulated hepatic SREBP-1c cleavage (Zhang et al., 2018). This would be confirmed by the fact that CEL decreased the expression of liver protein SREBP-1c, while the *Srebf1* mRNA expression in HFD-induced steatotic liver remained unchanged (Zhang et al., 2016). CEL either did not affect or normalized significantly downregulated protein expression of maturated (active, cleaved, and nuclear) SREBP1-m in set 1 and set 2, respectively. Moreover, CEL had no additional effect on SREBP1 activity, which is calculated as the ratio of SREBP1-m to premature SREPBP1-p, which was significantly increased by feeding mice WD/FG in both sets. Therefore, we may summarize that CEL does not produce its ameliorative effect on MASH primarily through the modulation of SREBP1 activity under our *in vivo* experimental setting.

Due to the histological scoring system’s limitations and variability in set 1, we could not clearly demonstrate a difference between the early and late stages of the positive control MASH at weeks 16 and 20, respectively. However, at week 20 and later on (Arora et al., 2023), we observed a smaller, firmer liver with less fat, confirmed biochemically and microscopically, indicating disease progression characterized by the decreasing amount of steatotic hepatocytes, the disorganized microarchitecture, and the development of liver cirrhosis and tumors. Given the considerably inconsistent findings regarding histopathological outcomes, gene mRNA (namely, *Mtor* and *Galr2*), and oxidative stress across the experimental sets, both our research team and other scientists posit that MASLD, the transition from MASLD to MASH (i.e., early-stage MASH), and advanced MASH (i.e., later-stage MASH) represent distinct and complex phenotypes (Martin et al., 2021; Arora et al., 2023). These conditions appear to involve unique signaling pathways in the liver affected by CEL in a considerably different manner in early-stage and later-stage MASH, with CEL showing more beneficial effects in the earlier stage of MASH (i.e., set 1). For example, *Nrf2*, *Hmox1*, *Sod2*, and *Galr2* gene expressions showed variable responses to CEL in the liver. The heightened expression of antioxidant molecules was attributed to their defensive and adaptive response against the substantial reactive oxygen species production and oxidative stress triggered by the Western diet. Moreover, the same study demonstrated that mitochondria generate less cellular oxidative stress, resulting in decreased TBARS at the stage of MASH formation (Staňková et al., 2021). Another intriguing study investigated the effects of a prolonged HFD on mRNA expression across a critical set of genes (including metabolic and antioxidant ones) in C57BL/6J mice. Although SREBP expression remained unchanged in this mouse strain, PGC-1α decreased and Nrf2 showed significant enhancement, mirroring our findings (Kim et al., 2004). Similarly, TBARS, conjugated dienes, nitrites, and gene expression of *Nrf2*, *Hmox1*, and also slightly of *Sod2* were concomitantly upregulated by WD/FD with down-regulating or, more appropriately, normalizing the effect of CEL at the end of set 1. On the other hand, CEL enhanced gene expression of antioxidant molecule genes in the situation when they were induced by long-term feeding with the Western-type diet at a much lower extent accompanied by decreasing oxidative stress in the liver of positive control mice at set 2. CEL has been found to activate SOD2 and Nrf2-HO1, contributing to its hepatoprotective effects in the liver of HFD obese mice (Luo et al., 2017; Zhang et al., 2016; Li M. et al., 2022). Moreover, CEL was shown to induce HO-1 through the upregulation of Nrf2 in hepatoma cells (Tseng et al., 2017). In our study, CEL significantly decreased both the liver inflammatory score and pro-inflammatory gene expression (*Tnfa* and *Il1b*) at the end of week 16, similar to that in various other studies with mouse models of fatty liver (Zhang et al., 2016; Li M. et al., 2022). Notably, macrophage infiltration and expression of macrophage M1 biomarkers (e.g., IL-6, IL-1β, TNF-α, and iNOS mRNA) were decreased after 3 weeks of CEL treatment in the livers of C57BL/6 mice induced with HFD (Luo et al., 2017), which can, at least partially, explain our findings. Wang et al. (2020) also demonstrated potent anti-inflammatory properties of CEL that remarkably suppressed the protein levels of pro-inflammatory cytokines (IL-1β, IL-6, IL-18, and TNF-α) while increasing the levels of anti-inflammatory cytokines (IL-10 and IL-13) in the serum and fibrotic livers of rats in a dose-dependent manner, while the lowest daily dose of 250 μg/kg CEL was not significantly effective. The relatively low CEL dose used in our study could explain why we did not achieve a significant reduction in mildly increased serum and liver TNF-α with CEL administration. Finally, our results on the anti-inflammatory effect of CEL in the early stage of MASH could be supported by the study demonstrating that transcription analysis provided a more promising means for identifying an immune profile as there are large discrepancies and lack of correlation between transcription and protein expression data when using the multiomics approach (Jacobsen et al., 2024). Taken together, we suppose that CEL is less effective on late-stage MASH in the second set of our *in vivo* experiments because other molecular signaling cascades and genes are involved in this stage of the disease.

The involvement of galanin in liver fibrosis and inflammation is multifaceted, with varying research findings (He et al., 2023). Galanin was found to exert its anti-inflammatory effects primarily through these receptor subtypes, particularly GalR2/3, while GalR1 is believed to be pro-inflammatory and pro-fibrogenic in the liver (Lang and Kofler, 2011; Mills et al., 2021; He et al., 2023). The hepatic expression of the *Galr2* gene, which was the only detected member of the galanin family at measurable mRNA levels in the mouse liver, was also variable throughout our study. Recently, it was demonstrated that human patients with MASLD have increased levels of serum galanin and that daily 5-week-lasting treatment with CEL ameliorates HFD/high cholesterol-induced MASH in mice (He et al., 2023). Moreover, in the same study, it was shown that murine macrophages express GalR2, proposing a new role for galanin in inhibiting the pro-inflammatory phenotype of macrophages and inducing their M2-polarization (He et al., 2023). Notably, the same authors revealed that galanin can inhibit primary rat hepatic stellate cell (HSC) activation and suppress the pro-fibrogenic characteristics of HSCs by activating the GalR2 receptor, whose expression is induced in activated HSCs but undetectable in quiescent HSCs (He et al., 2016). In our *in vitro* experiment, celastrol did not reduce palmitic acid-induced lipotoxicity in primary hepatocytes even at the highest tested non-toxic concentration. The selective GalR2 inhibitor, M871, significantly enhanced PA-induced cytotoxicity and nitric oxidative stress alongside rather reduced lipid accumulation in hepatocytes. M871 showed its tendency to reverse the effects of CEL. These results suggest that GalR2 plays an important role in hepatoprotection independent of lipid accumulation and that, in the liver, CEL might positively affect GalR2 expression in cells other than primary (untransformed) hepatocytes (e.g., macrophages/Kupffer cells, HSCs, fibroblasts, and hepatoma cells), at least in the early stages of MASH. Spexin-mediated mitigating effects on obesity and MASLD in mice on HFD and in PA-induced HepG2 cells were eradicated by M871, proving that spexin, a known GalR2/3 agonist, produces its beneficial effect through the activation of GalR2 (Wang et al., 2022). Moreover, anti-apoptotic and anti-proliferative effects of galanin and GALP were shown to be mediated through GalR2 (Berger et al., 2004; Tofighi et al., 2008), which can explain no liver cancer development in mice under the CEL treatment. Interestingly, CEL in our study also exerted highly similar ameliorative effects as spexin *in vivo*, including changes in *Ppargc1*, *Fasn*, *Srebf1*, and *Sirt1* expressions (Wang et al., 2022). Therefore, we can speculate that CEL could exert its MASH ameliorative effects through increased GalR2 gene and protein expression in the liver, namely, in set 1 of our *in vivo* experiments. Interestingly, fatostatin did not affect liver *Galr2* gene expression, suggesting that SREBP activity has no effect on *Galr2*/GalR2 expression and *vice versa* in the liver. The liver activity of SREBP1 in our *in vivo* experimental settings both did not correlate with GalR2 protein expression and was not influenced by CEL.

Notably, the distribution of galanin receptor subtypes in rodents differs: GALR1 is exclusively expressed in the central and peripheral nervous systems, while GALR2 and GALR3 are widely distributed in both the central and peripheral tissues of rats (Mills et al., 2021; Waters and Krause, 2000). Previously, the expression of the Galr1 gene was detected in the brain, spinal cord, heart, and skeletal muscle; however, no mouse *Galr1* mRNA was detected in the liver, kidney, testis, lung, and spleen (Wang et al., 1997b). According to RNA profiling data sets generated later by the Mouse ENCODE project, there is some extent of expression of *Galr2*, negligible expression of *Galr3,* and no expression of *Gal, Galp*, or *Galr1* RNAs in the adult liver and heart under physiological conditions of 8-weak-adult C57BL/6J mice. Interestingly, *Galp* and *Galr3* were revealed in embryonal liver but not in adult ones, and all three *Galr* RNAs were detected in adult genital fat pads, with the most pronounced one being *Galr2* (Yue et al., 2014; National Library of Medicine–National Centre for Biotechnology Information, 2024). ENCODE data showed GalR2 to be the only receptor subtype in adult mouse hearts, cardiomyocytes, and H9C2 cardiomyoblasts (Boal et al., 2022). In terms of *Galr2* gene expression, our observations align with the highest levels of its mRNA detected in genes related to the galanin system, both in the liver and heart of adult C57BL/6J mice, regardless of their age. Conversely, we observed an age-related increase in gene expressions of *Gal*, *Galr1*, and *Galr3* (in order of decreasing quantity) in the heart ventricles, with a peak occurring between weeks 16 and 20 on the controlled STD. According to the available literature, *Gal* and *Galr3* expressions have not been detected in the mouse heart until the completion of this manuscript. Furthermore, we had originally demonstrated that WD/FG significantly increased mRNA levels of *Gal*, *Galr1*, *Galr2*, and *Galr3* in mouse heart ventricles compared to those in negative controls in our experiments, suggesting that other members except for GalR2 might play a role in cardiovascular physiology and pathophysiology related to metabolic diseases. We had previously described the gene and protein expressions of galanin, GALP (Škopek, 2013), and galanin receptors (GalR1, GalR2, and GalR3) in rat hearts and their modulation under stress (Škopek, 2013; Šípková et al., 2017b). Moreover, galanin’s cardioprotective and crucial role in regulating cardiac autophagy and apoptosis in hypertrophied hearts, following myocardial infarction in mice, was demonstrated (Martinelli et al., 2021). A recently published review based on the experimental data on many studies summarized that galanin plays a role in the development of insulin resistance and diabetic heart conditions; however, it also helps alleviate hyperglycemia and improves insulin sensitivity, enhances glucose utilization, and promotes mitochondrial growth in the cardiac muscle through GalR2. These findings depict GalR2 as a potential therapeutic target for treating diabetic cardiomyopathy (She et al., 2023). Moreover, genetic suppression of *Galr2 in vivo* (by using *Galr2* knockout mice) and *in vitro* (by using siRNA transfection) promoted cardiac hypertrophy, fibrosis, and mitochondrial oxidative stress and eradicated the beneficial effects of galanin on mouse heart and primary cardiomyocytes, respectively. Therefore, the authors concluded that targeting GalR2 might offer novel therapeutic strategies for heart diseases (Boal et al., 2022). Stimulation of GalR2 signaling by selective galanin ligands was also presented to be involved in the attenuation of myocardial ischemic/reperfusion injury and the improvement of cardiac function in rats *in vivo*, which was completely reversed by M871 (Palkeeva et al., 2019; Serebryakova et al., 2023). In our *in vivo* experiments, CEL did not affect both *Gal* and *Galr2* expressions that were upregulated by WD/FG, probably as a result of an adaptive mechanism to metabolic stress, thus preserving the protective effect of locally produced galanin on the heart through GalR2. Moreover, CEL was histologically demonstrated to be safe not only for the heart but also for the other major organs including the liver, spleen, lungs, kidneys, and brain of HFD-induced obese mice (Fan et al., 2022).

Contrarily to the stimulation of GalR2, which activates phospholipase C-induced signaling pathways, GALR1 and GALR3 primarily decrease cAMP-mediated signaling pathways, hence interposing the inhibitory effects of galanin (Šípková, 2017a). The data on the role of GalR1 and GalR3 in heart diseases are controversial or missing (She et al., 2023). We present evidence of GalR1 expression in the mouse heart, both at the mRNA level and at the protein level detected by immunohistochemistry. In our scenario, the failure of immunostaining for the other proteins of interest (i.e., GalR2 or GalR3) might be attributed to the fixation of heart tissue by paraformaldehyde or/and the possibility that, under the experimental conditions, the endogenous GalR2 and GalR3 adopted a unique configuration different from the immunogens used to generate the antibodies (Lu and Bartfai, 2009; Michalickova et al., 2023). In general, there is a lack of sensitive and selective antibodies capable of recognizing specific endogenous G-protein-coupled receptors like mouse GalRs (Lu and Bartfai, 2009), although previously we managed to detect immunoreactivity of all three types of receptors in rat heart sections using the completely same antibodies against mouse GalRs (Šípková et al., 2017b). Therefore, when screening the effect of different modalities on galaninergic receptors, gene expression analysis is preferably used. In our experiment, GalR1 immunoreactivity was positive in cardiomyocytes of both heart atria and ventricles and was concentrated in the intercalated discs, mimicking our previous findings in rat hearts (Šípková et al., 2017b). Intercalated discs serve as intricate structures that facilitate both mechanical and electrical connections between cardiomyocytes, showing that galanin collaborates with other transmitters to regulate cardiac function as previous research indicates that galanin, through its interaction with GalR1, decreases acetylcholine release and suppresses vagal bradycardia (Herring et al., 2012). The use of the preferable GaR1 antagonist M40 resulted in enhanced cardiac function and reduced remodeling in rats with heart failure. The potential mechanism underlying this cardioprotective effect involves upregulation of cardiac SERCA2 (sarco/endoplasmic reticulum calcium ATPase 2) and a decrease in plasma IL-6 (Chen et al., 2015). Moreover, we had previously detected both mRNA and immunoreactivity of GalR1 in endothelial cells and discussed that utilizing GalR1 antagonists in clinical settings might yield antiangiogenic effects to counteract detrimental angiogenesis (Michalickova et al., 2023).

Another review primarily outlined the favorable impacts of CEL on cardiovascular disease (CVD), derived from *in vitro* and *in vivo* preclinical research and potential underlying mechanisms, including CEL´s inhibitory effect on the central galaninergic system (Li Z. et al., 2022). As CEL in our study significantly reduced *Galr1* gene expression in the heart ventricle, we can assume that it can contribute to CEL’s overall favorable metabolic effect and possibly also the cardioprotective effect. This pattern of CEL can be at least partially (via decreasing fat intake, body weight, and adiposity) related to the modulation of SREBP activity as FAT produced the same Galr1 down-regulating effect in the mouse heart. It was demonstrated *in vitro* that mouse *Galr1* gene expression is upregulated by cAMP through a CREB-dependent mechanism (Zachariou et al., 2001) and that CREB is a co-activator of SREBP-mediated transcription of reporter genes (Oliner et al., 1996). These and our findings (i.e., enhanced *Galr1* expression in hearts of obese mice on WD/FG) suggest that increasing activation of SREBPs by HFD in obese mice (Soyal et al., 2015; Zhang et al., 2016) can induce *Galr1* gene expression in the heart. It seems to be favorable that CEL does not completely suppress the *Galr1* gene and GalR1, as we showed, because mice with a completely knockout (KO) *Galr1* gene have modified intake of only HFD (not STD) and experience glucose metabolism impairment, while *Galr1* heterozygotes do not differ from wild-type mice (Zorrilla et al., 2007). Interestingly, both changes in food behaviors under challenging HFD and glucose intolerance were also described for *Gal*-KO mice (Ahrén et al., 2004; Adams et al., 2008), suggesting the role of galanin and GalR1 in adjusting food intake and metabolic responses to variations in dietary fat while also influencing glucose regulation in mice (Zorrilla et al., 2007).

On the contrary, the *Galr3*-KO (backcrossed on the C57BL/6 background) mouse strain displayed physiological breeding and physical development alongside a similar trend (n.s.) of higher plasma TG and cholesterol levels compared with age-matched wild-type mice. Notably, male *Galr3*-KO mice exhibited an anxiety-like phenotype and reduced social affiliation (Brunner et al., 2014). However, the role of GalR3 in CVDs remains unclear as the expression of Galr3 is generally very low (i.e., as in our study) or missing depending on the age, tissue, and species, as we discussed above. Furthermore, CEL significantly increased mouse heart *Galr3* expression only when it was not upregulated in positive controls. On the other hand, FAT significantly downregulated WD/FG-enhanced *Galr3* mRNA, like in the cases of *Gal*, *Galr1*, and *Galr*2 genes, indicating the role of SREBP and fat metabolism in the gene expression of members of the galaninergic system in the heart of obese mice. To the best of our knowledge, this significant downregulating effect of FAT on the galaninergic system in the heart has not yet been described anywhere. However, both SREBP1 and GalR1/GalR2 played pivotal roles within the adipogenic signaling pathways induced by the HFD in mice. Notably, cinchonine, a natural compound, exerted simultaneous inhibitory effects on these pathways (i.e., it significantly downregulated gene expression of *Srepbf1*, *Galr1*, and *Galr2* in fat tissue), resulting in reduced adipogenesis and mitigated obesity (Jung et al., 2012). However, the specific regulatory relationship between SREBPs and the galaninergic system remains unclear based on the current research. Therefore, more extensive studies are required to elaborate on the SREBP–galaninergic system interactions in various organs (including the heart) and species. Taken together, we can assume that the peripheral effects of CEL on the heart are produced mainly by decreasing the expression of *Galr1* in WD/FG-induced obese mice. It can be supported by the fact that CEL was likewise demonstrated to both effectively inhibit fat intake and promote weight loss by downregulating the expression of galanin and its receptors (specifically GalR 1 and 3) in the mouse hypothalamus during HFD conditions (Fang et al., 2019).

## 5 Conclusion

According to our results, CEL may have a beneficial effect on the galaninergic system modulation in the heart and liver of obese male mice on the Western-type atherogenic diet. Along with reducing the fat intake, weight gain, and amelioration of MASLD to MASH progression in mice, CEL also affected the expression of galanin and its receptors in the heart ventricles and liver lobes, which may be involved in the regulation of energy metabolism, oxidative stress, and inflammation. Therefore, CEL may be a potential therapeutic agent for these metabolic dysfunction-associated disorders in humans. However, the exact mechanisms and implications of celastrol’s action on the galaninergic system are not fully understood and require further investigation. Future research should also evaluate its impact on female animal models of MASLD/MASH, particularly those with deficits or alterations in sex hormone levels, highlighting the need for personalized treatment approaches for women.

## Data Availability

The raw data supporting the conclusions of this article will be made available by the authors, without undue reservation.
