# Supplementary material for: Effects of celastrol on the heart and liver galaninergic system expression in a mouse model of Western-type diet-induced obesity and metabolic dysfunction-associated steatotic liver disease and steatohepatitis
Source: Front Pharmacol. 2025 Feb 4;16:1476994. doi: 10.3389/fphar.2025.1476994 (PMC11832397; doi:10.3389/fphar.2025.1476994)
Supplement: Supplementary file 1 [file DataSheet1.docx]

# **Supplementary materials**

# **Supplementary methods**

## ***In vitro* MASLD model**

Unless otherwise stated, all the high-quality chemicals were purchased from Sigma-Aldrich (Merck, Germany) or P-Lab (Czech Republic).

## **Primary rat hepatocyte culture**

Adult male Wistar rats (Velaz, Lysolaje, Czech Republic) with weights of 280-300 g and ages of 8-10 weeks of age were used following a well-established method of the palmitic acid-induced *in vitro* MAFLD model as we described in details previously (Arora et al., 2023). Shortly, primary hepatocytes isolated by standard two-step collagenase (crude collagenase, Sevapharma, Czech Republic) method were suspended in a complete medium (William’s E medium supplemented with 100 IU/ml penicillin, 100 µg/ml streptomycin, 2.5 μg/mL amphotericin B,6 μg/mL (0.06%) insulin, 2 mM L-glutamine, 5% (v/v) fetal bovine serum (FBS)) and plated on collagenated (using collagen obtained from rat tail tendon Cat. No. 11179179001, ROCHE through Merck) 24-well plates (24-WP) and 6-well plates (6-WP) at a density of 1x10^6^ of viable cells per 1.5 ml. Following the cell attachment and changing of the medium, the hepatocytes were cultured overnight. Firstly, celastrol (CEL, Tripterin, # HY-13067) and M871 (GalR2 inhibitor, # HY-P1130, both from MedChemExpress, USA, through Scintila, Czech Republic) were dissolved in DMSO so as to prepare a stock solution. The fresh complete medium with test substances at nontoxic concentrations (CEL 500 nM, M871 100 nM, CEL+M871) or DMSO (final concentration 0.125%) were then applied to the cells and incubated for 6 hours as pre-treatment prior to the application of palmitic acid (PA, 1 mM) for a further 18 hours. The PA was firstly dissolved in absolute ethanol and subsequently in the complete medium containing 1% FBS and 2% BSA under sonication. At the end of the *in vitro* experiment, the medium was sampled for biochemical analysis purposes (alanine aminotransferase, ALT; nitrites measurement assays). The attached hepatocytes were washed twice with cold (4°C) phosphate buffered saline (PBS) to be used either for the Oil Red O staining or the viability assay employing the MTT test, as described below.

- 1. **MTT test**

The standardized colorimetric MTT (3-(4, 5-dimethylthiazolyl-2)-2, 5-diphenyltetrazolium bromide) assay was performed using tetrazolium salt to measure cell viability (e.g. metabolic activity) or cytotoxicity (Arora et al., 2023). The results were expressed in percentages of the average control absorbance at 540 nm, whereby the control had 100% viability. Moreover, the MTT test, together with the ALT release from the hepatocytes into the culture medium, was used to assess the optimal dose of the PA and non-toxic concentrations of celastrol and M871 suitable for our *in vitro* study.

- 1. **Determination of the alanine aminotransferase release and nitrites production**

A commercial ALT kit (# 10452, BioVendor, Czech Republic) was used for the manual determination of the catalytic concentration of ALT [IU/l; 1 μkat/l = 60 IU/l] in the cultured hepatocyte medium.

In order to analyze the oxidative stress, the production of nitric oxide (NO) was measured applying an indirect spontaneous oxidation to nitrites and negligible nitrates method. The amount of NO_2_^-^ ions, the main stable product following NO oxidation in the medium, was transferred to colored diazonium salt using the Griess reagent (1% sulfanilamide, 0.1% naphtylethylendiamine, 2.5% trihydrogenphosphoric acid) and subsequently measured spectrophotometrically at 540 nm. The nitrite concentration [µM] was subtracted from the NaNO_2_ standard curve (Arora et al., 2023).

- 1. **Oil Red O staining**

Oil Red O, also known as Solvent Red 27, is a fat-soluble dye that is used for the staining of neutral triglycerides and lipids within formalin-fixed cells. Staining (with the omission of the hematoxylin staining step) was performed according to the instructions provided by the manufacturer (# MAK194, Sigma-Aldrich through Merck, Germany) in order to visualize the accumulation of PA in the hepatocytes. The stained hepatocytes were covered with water so as to avoid their dehydration and were viewed under a light inversion microscope through a phase contrast filter, whereby the lipid droplets inside the cells appeared in orange-red (Arora et al., 2023). Finally, the stain was extracted in 100% isopropanol for quantification and measurement of Oil Red O stain in a 96 well plate reader at 492 nm.

1. **Determination of the TNF-α**

The quantitative detection of the mouse TNF-α was performed using high-sensitivity ELISA kits for serum TNF-α (Invitrogen through Thermo Fisher Scientific, Waltham, Massachusetts, USA) and liver homogenate TNF-α (Abcam through iBioTech, Czech Republic). The concentration in pg/ml was calculated from the standard calibration curve according to the manufacturer’s instructions. Liver TNF-α was expressed in pg/ng of protein which was determined by the Pierce™ BCA Protein Assay Kit (Thermo Scientific™).

# **Supplementary results**

**Figure S1**


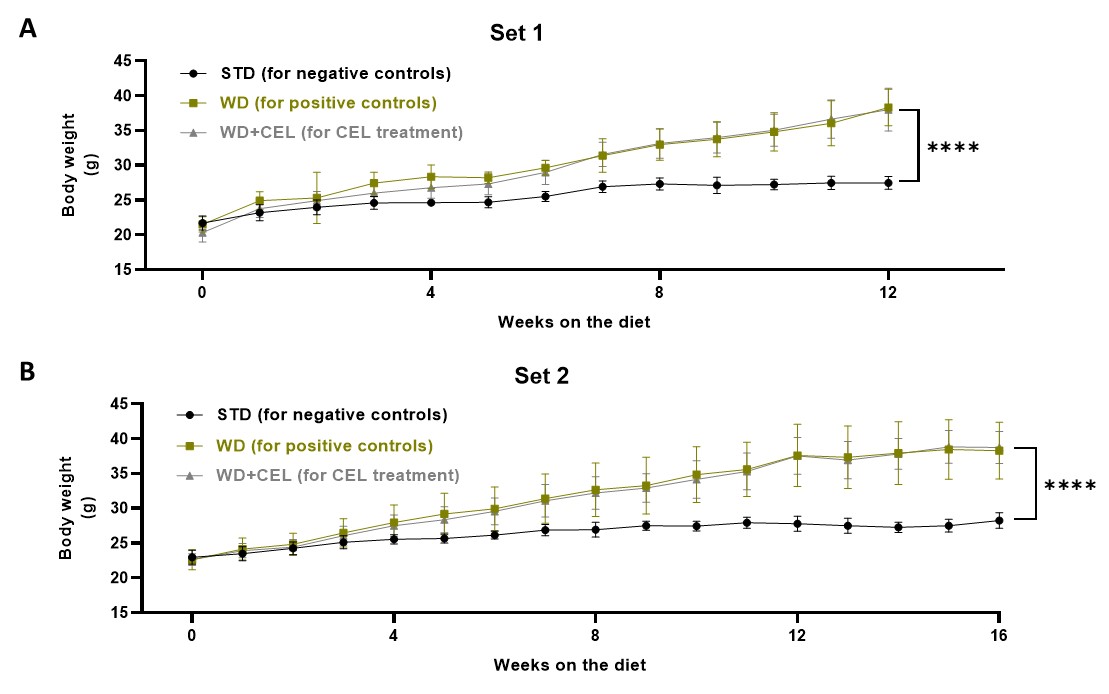


**Figure S1:** Effect of the Western-type diet on mice body weights in each set of *in vivo* experiments. **A**: Weekly body weights of the experimental set 1 mice during 12 weeks on the diet. **B**: Weekly body weights of the experimental set 2 mice during 16 weeks on the diet. Data are expressed as means ± SD (n = 3 for standard diet, STD, groups of negative controls; n = 7-8 for Western-type diet, WD, groups of positive controls and CEL treatments, WD+CEL, as assessed in Table 1), where ****p<0.001 when comparing respective WD or WD+STD groups against STD group as assessed by two-way ANOVA with post hoc Bonferroni test.

**Figure S2**


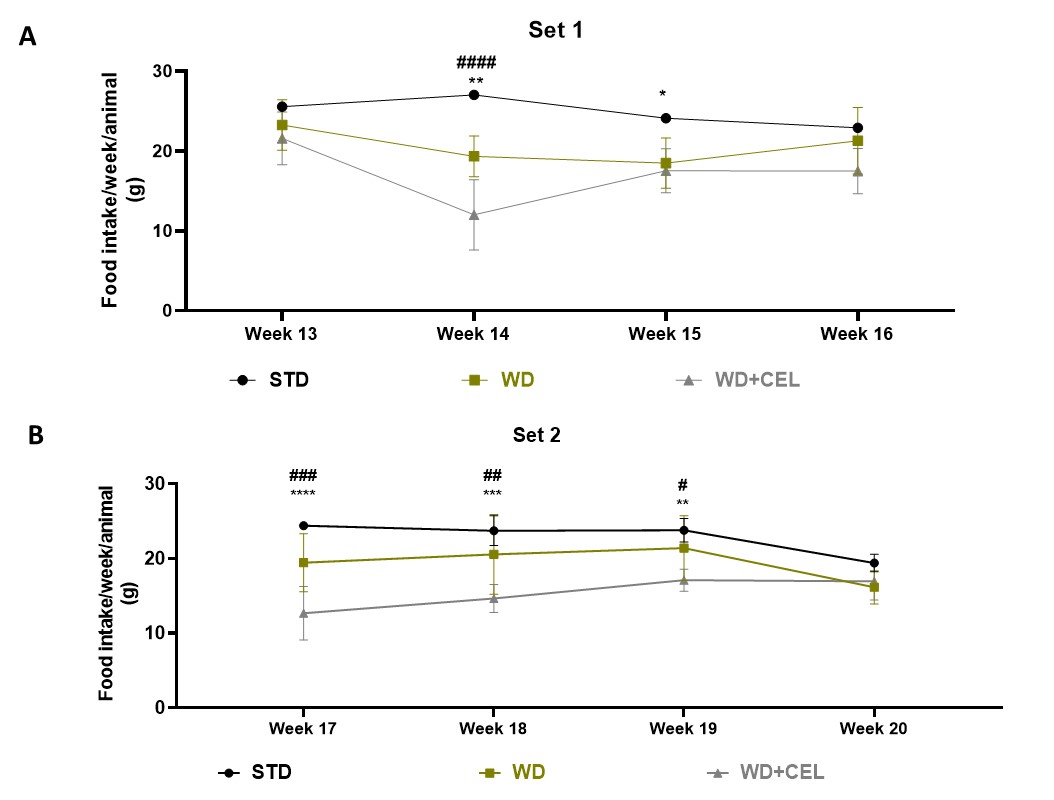


**Figure S2:** Effect of 4-week celastrol (CEL) treatment on food consumption. **A**: Final weekly (measured during weeks 12-16) food consumption per mice of set 1. **B**: Final weekly (measured during weeks 16-20) food consumption per mouse of set 2. Data are expressed as means ± SD (n = 3 for negative controls, n = 7-8 for positive controls and CEL treatment as stated in Table 1), where **p<0.01, ***p<0.001, ****p<0.001 when comparing respective positive control (WD) against negative control (STD), and ^##^p<0.01, ^###^p<0.001, ^####^p<0.001 when comparing CEL treatment (WD+CEL) against positive control (WD) as assessed by two-way ANOVA with post hoc Bonferroni test.

**Figure S3**


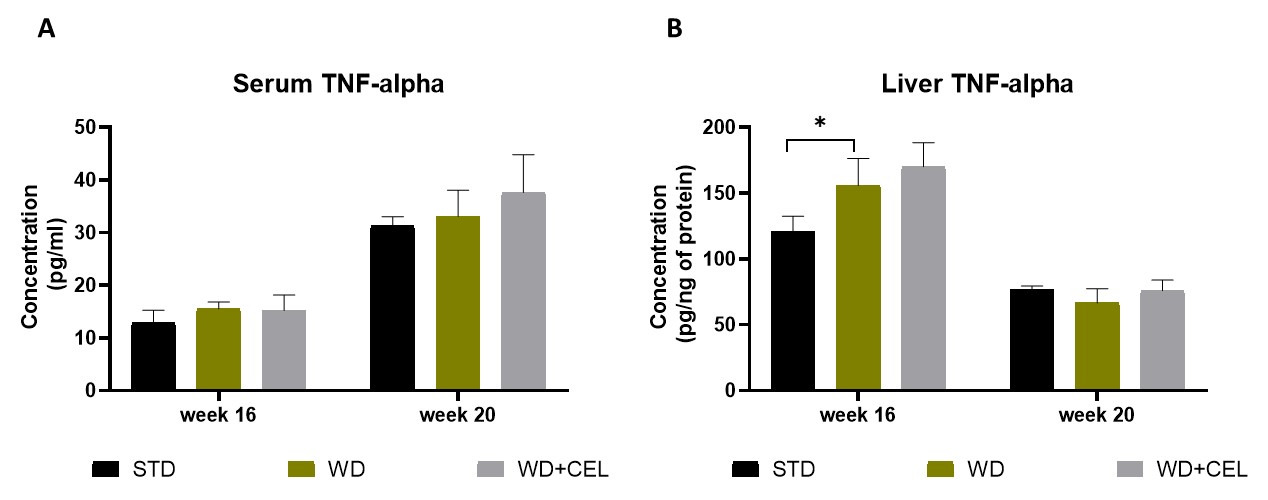


**Figure S3:** Effect of celastrol treatment (WD+CEL) on concentrations of mice serum TNF-α **(A)** and liver TNF-α **(B)** in both sets of *in vivo* experiment. Data are expressed as means + SD (n = 3 and 7-8/group as noted in Table 1), *p<0.05 when comparing respective positive control (WD) against negative control (STD) as assessed by one-way ANOVA with post hoc Bonferroni test.

**Figure S4**


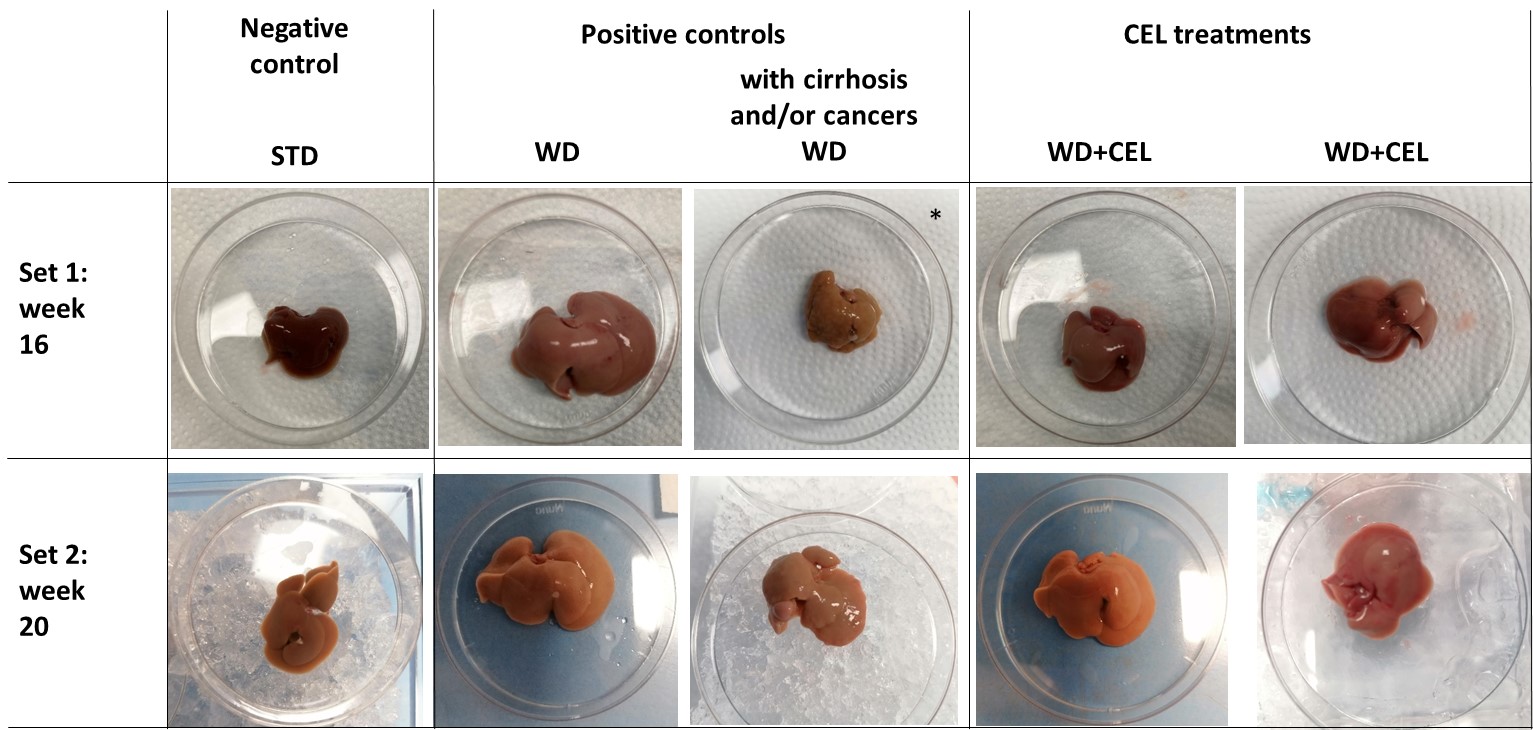


**Figure S4:** Example photographs of livers sampled from mice of positive control (STD), negative control (WD), and CEL treated (WD+CEL) groups at the end of week 16 (set 1) and week 20 (set 2). All isolated livers were transferred to a 60 mm diameter petri dish. Note: One mouse suffering of severe liver cirrhosis (presented in the image*) with hyperbilirubinemia and a cyst of the right kidney was excluded from all evaluations.

**Figure S5**


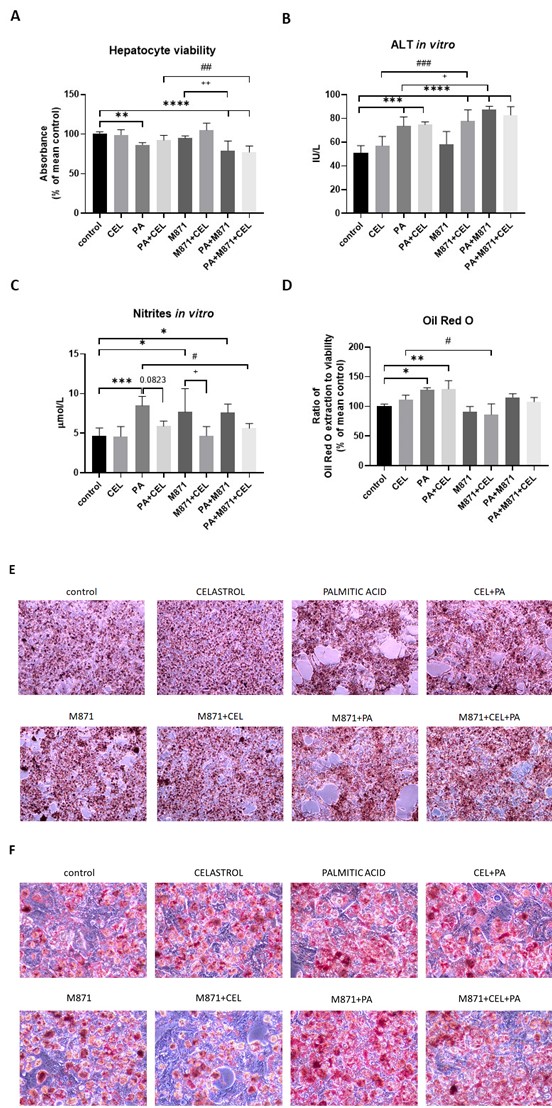


**Figure S5:** The impact of celastrol (CEL) and GalR2 inhibitor, M871, treatments on palmitic acid (PA)-induced lipotoxicity in the primary hepatocytes. **(A)** Hepatocyte viability as determined by the MTT test. **(B)** The catalytic concentration of ALT (alanine aminotransferase) released into the culture medium. **(C)** The cell culture medium concentration of nitrites produced by the hepatocytes. **(D)** Relative quantification of Oil Red O stain extracted from hepatocytes. All the data are expressed as the means + SD of 2 independent experiments (n = 6 for A, B, C, and n = 4 for D): **p<0.01, ***p<0.001, ****p<0.0001 when compared to the control hepatocyte culture; ^#^p<0.05, ^##^p<0.01, ^###^p<0.001 when compared to either PA+CEL (A), CEL (B, D) or PA group (C); ^+^p<0.05, ^++^p<0.01 when compared to M871 (A, C) or PA group (B) as assessed by one-way ANOVA with the post hoc Bonferroni test. **(E, F)** The Oil Red O staining which was conducted to qualitatively evaluate cell morphology, size, fat quantity, and distribution in hepatocytes of the respective groups, as depicted in the sample images visualized by a light inversion microscope with phase contrast at a magnification of 100x (E) and 400x (F). An inherent fat content was observed in hepatocytes of the control, CEL and M871 groups as the complete media containing at least 1% FBS was used. Cell cultures exposed solely to PA exhibited significantly less surviving hepatocytes, characterized by a high number of red oil droplets, indicating enhanced intracellular fatty acid absorption. Pre-treatment with CEL prior to PA slightly augmented the number of viable hepatocytes but had no effect on fat content in lipid-laden hepatocytes. Interestingly, M871 decreased lipid accumulation, namely in combination with CEL.
